# Investigating the effects of a daily multidisciplinary intensive outpatient rehabilitation program on innovative biomarkers in people with Parkinson’s disease: Study protocol for a phase III randomized controlled clinical trial

**DOI:** 10.1371/journal.pone.0309405

**Published:** 2024-10-23

**Authors:** Francesca Lea Saibene, Cristina Agliardi, Anna Salvatore, Pietro Arcuri, Anna Castagna, Silvia Gobbo, Federico Merlo, Thomas Bowman, Denise Anastasi, Chiara Pagliari, Elisabetta Farina, Margherita Alberoni, Elena Calabrese, Francesca La Rosa, Chiara Arienti, Marina Saresella, Franca Rosa Guerini, Davide Cattaneo, Francesca Baglio, Mario Clerici, Jorge Navarro, Mario Meloni

**Affiliations:** 1 IRCCS Fondazione Don Carlo Gnocchi ONLUS, Milan, Italy; 2 Department of Biomedical Sciences, University of Sassari, Sassari, Italy; 3 Department of Physiopathology and Transplants, University of Milan, Milan, Italy; 4 Neurology Unit, Azienda Ospedaliero-Universitaria, Cagliari, Italy; Philadelphia VA Medical Center, UNITED STATES OF AMERICA

## Abstract

**Background:**

To date, there has been no medication that has prevented the progression of Parkinson’s disease (PD). Many benefits of intensive and multidisciplinary rehabilitation program for PD are supported by clinical, epidemiological, and experimental data. The main question is whether high-intensity motor and cognitive exercises have an effect on the disease’s biological mechanisms.

**Objective:**

This study protocol is a Randomized Controlled Trial (RCT) designed to determine the efficacy of an experimental, intensive, and multidisciplinary treatment in comparison to a home-based self-treatment in improving biomolecular and functional parameters in PD.

**Methods:**

A total of 72 participants will be randomly allocated to two different groups, experimental (n = 36) and control group (n = 36). The rehabilitation program will last 6 consecutive weeks and will involve the execution of a total of 30 sessions, one for each day of the week from Monday to Friday. Participants allocated to the control group will carry out a home-based self-treatment program that includes muscle-stretching and active mobilization exercises for 40’/day for 6 consecutive weeks. The primary outcome measure is the effects of both treatments on a new set of molecular biomarkers such as oligomeric alpha-synuclein and neurotrophic factors measured in peripheral neural derived extracellular vesicles (NDEVs). Secondary outcomes will include changes of motor and non-motor symptoms, balance and gait performance and cognitive functioning. This RCT has been registered as “Intensive Multidisciplinary Rehabilitation and Biomarkers in Parkinson’s Disease” on 30 May, 2022 to ClinicalTrials.gov with the Study ID number: NCT05452655.

**Discussion:**

This rehabilitation program is believed to be crucial in modifying biomolecular and functional parameters in people with PD. We expect that this study will provide additional evidence to understand the impact of an aerobic and intensive rehabilitation program on brain plasticity in patients with PD.

## Introduction

Parkinson’s Disease (PD) is a chronic and slowly progressive neurodegenerative disease, and its prevalence is on the rise at a faster pace compared to other neurological disorders [1]. PD is characterized by a wide range of both motor and nonmotor symptoms (NMS).

Rigidity, bradykinesia, resting tremor are common motor symptoms in PD arising in the early stages of the disease and are mostly influenced by the degeneration of dopaminergic neurons in substantia nigra pars compacta [2]. PD symptoms are now acknowledged to be more heterogeneous. Motor and non-motor features like cognitive impairment, sleep disorders, autonomic dysfunction, pain, fatigue, psychiatric symptoms, and olfactory dysfunction contribute to worsening the disability [3, 4] and the quality of life [5, 6].

In the literature, consistent evidence supports the use of levodopa and dopamine agonists as the principal treatment for motor symptoms [7]. However, despite pharmacological treatment, a majority of people with PD (PwPD) keep experiencing a broad spectrum of motor and nonmotor symptoms [8, 9]. In this context, several other therapies have been shown effective on other symptoms, even though evidence is less conclusive for PD treatments both in the early and advanced stages of the disease [10]. In light of this, an expanding body of research is documenting non-pharmacological treatments for PD symptomatology, including rehabilitation strategies [11, 12]. Even though a recent review reports the absence of statistical significance on the effect of multidisciplinary treatment over conventional treatment [13], a large body of scientific literature seems to converge on the usefulness of multidisciplinary rehabilitation for managing PD. In particular, this type of treatment has been shown effective on measures of Quality of Life [14], measures of motor symptoms [15, 16], non-motor symptoms as cognition, anxiety, and depression [17] and functional measures [18]. Moreover, it is also true that the nature of multidisciplinary treatments differs highly between studies: they can be composed of physiotherapy and cognitive training [19], physiotherapy and occupational therapy [20] or performed by multidisciplinary teams [21]. This heterogeneity points to the need for new standardized and highly controlled studies on this topic.

A recent phase 2 clinical trial in de novo PD patients compared moderate (60%-65% maximum heart rate), high-intense (80%-85% maximum heart rate) endurance exercise and usual care 4 days per week for 6 months. The clinical outcome was 6-month change in UPDRS Part III. Compared to usual care, high-intensity treadmill exercise resulted in fewer motor changes. Interestingly, the high-intensity group experienced an increase in maximal aerobic power ($\dot{V}o2\max$) compared with baseline [22].

In a recent single-center, double-blind, randomized controlled trial (Park-in-Shape) the authors aimed to evaluate the effectiveness of aerobic exercise delivered at home on relieving motor symptoms in PD subjects with mild disease severity. According to the authors, patients with mild disease severity can reduce off-state motor symptoms by performing aerobic exercise at home [23].

Non-pharmacologic treatment has not only been shown to affect symptomatology, but there is also preliminary evidence in favor of an effect of physical exercise on brain plasticity [24–26]. In particular, the effects of exercise could affect several plasticity-related aspects, among which synaptogenesis, angiogenesis, and neurogenesis [27, 28]. Results on animal models reveal that physical exercise causes changes in neurotrophic factors (NFs) [29–31]. On the same line, there are few studies in the literature linking the benefits of aerobic exercise in PwPD to the involvement of plasticity processes, such as increases in serum brain-derived neurotrophic factor (BDNF) [32] and BDNF-tyrosine receptor kinase B (TrkB) levels [33]. However, the paucity of studies in the literature and their methodological pitfalls do not allow solid conclusions on a potential increase of NFs levels in PD [26].

The absence of methodologically solid studies on the effect of multidisciplinary interventions and their effect on biomarkers points to the need for new standardized controlled randomized clinical trials on this topic.

Moreover, there is still a high level of heterogeneity among studies regarding the detection of α-synuclein as a main biomarker in PD patients [34]. For this reason, in addition to α-synuclein, we have included as a co-primary outcome a set of biological markers closely interconnected with α-synuclein in the PD pathological processes.

Our main research question was whether an integrated motor-cognitive aerobic rehabilitation approaches could affect the biological mechanisms underlying neurodegenerative processes in PD. Coherently, we decided to investigate the modifications of a new set of fluid-based PD biomarkers in response to an intensive rehabilitation program by a single-blind randomized controlled trial.

### Study objectives

The study aims to investigate the effect of an intensive, multidisciplinary, outpatient treatment protocol on a set of molecular biomarkers as well as on balance, gait, aerobic performance, cognitive functions, motor and non-motor symptoms in PwPD.

## Materials and methods

### Study design

The present study is designed as a randomized, controlled, two-arm trial with 1:1 allocation. It is a non-pharmacologically interventional study, and it compares a 6-consecutive weeks of daily outpatient intensive multidisciplinary rehabilitation treatment (Experimental Group, EXP-Group) to a 6-consecutive weeks of daily home-based self-treatment stretching program (Control Group, CTRL-Group) in PwPD.

**Fig 1:**
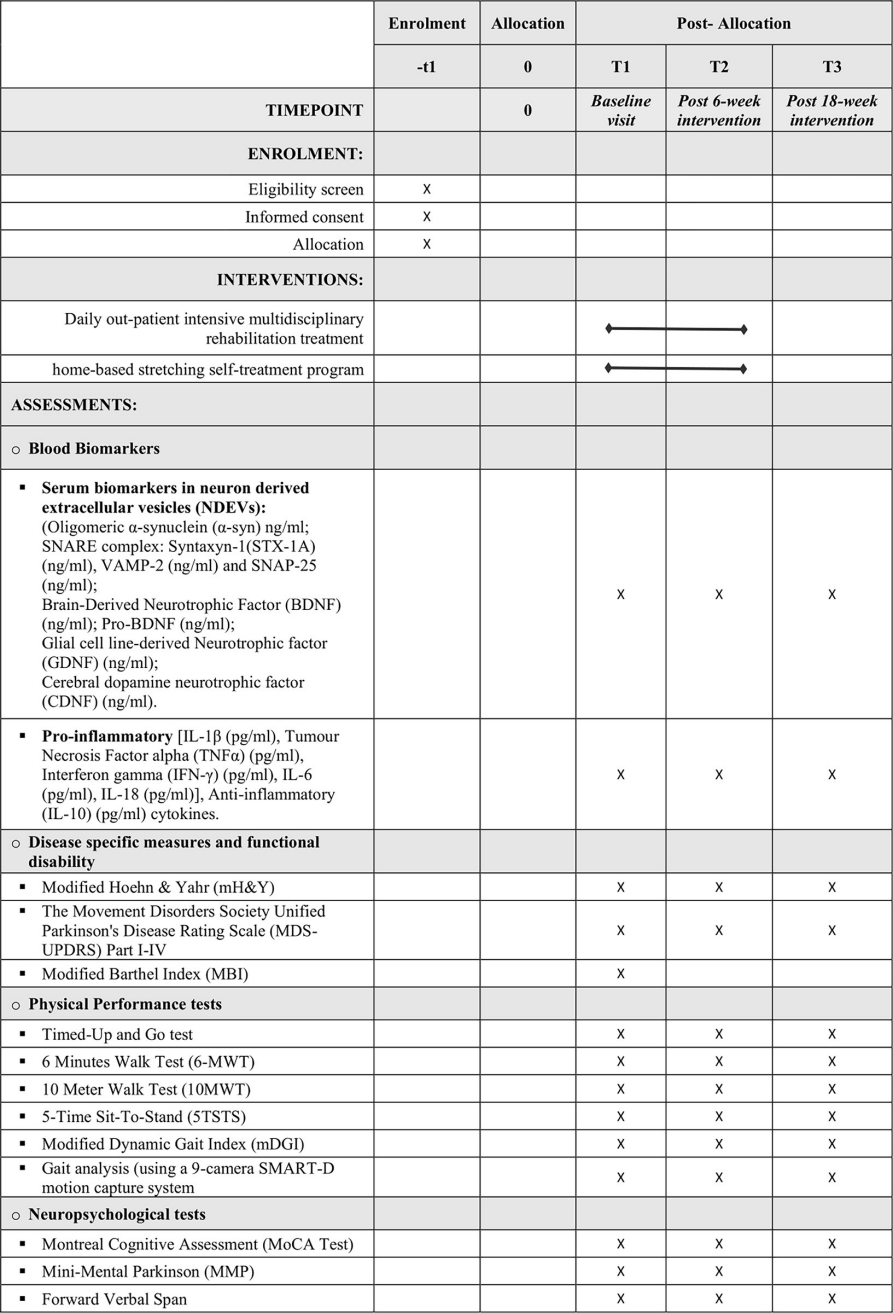
SPIRIT schedule of enrolment, interventions, and assessments.

A SPIRIT schedule of enrolment, interventions, and assessments can be found in Fig 1.

This protocol was constructed following the Standard Protocol Items: Recommendations for Interventional Trials (SPIRIT) checklist (see S1 Checklist) [35].

**Fig 2:**
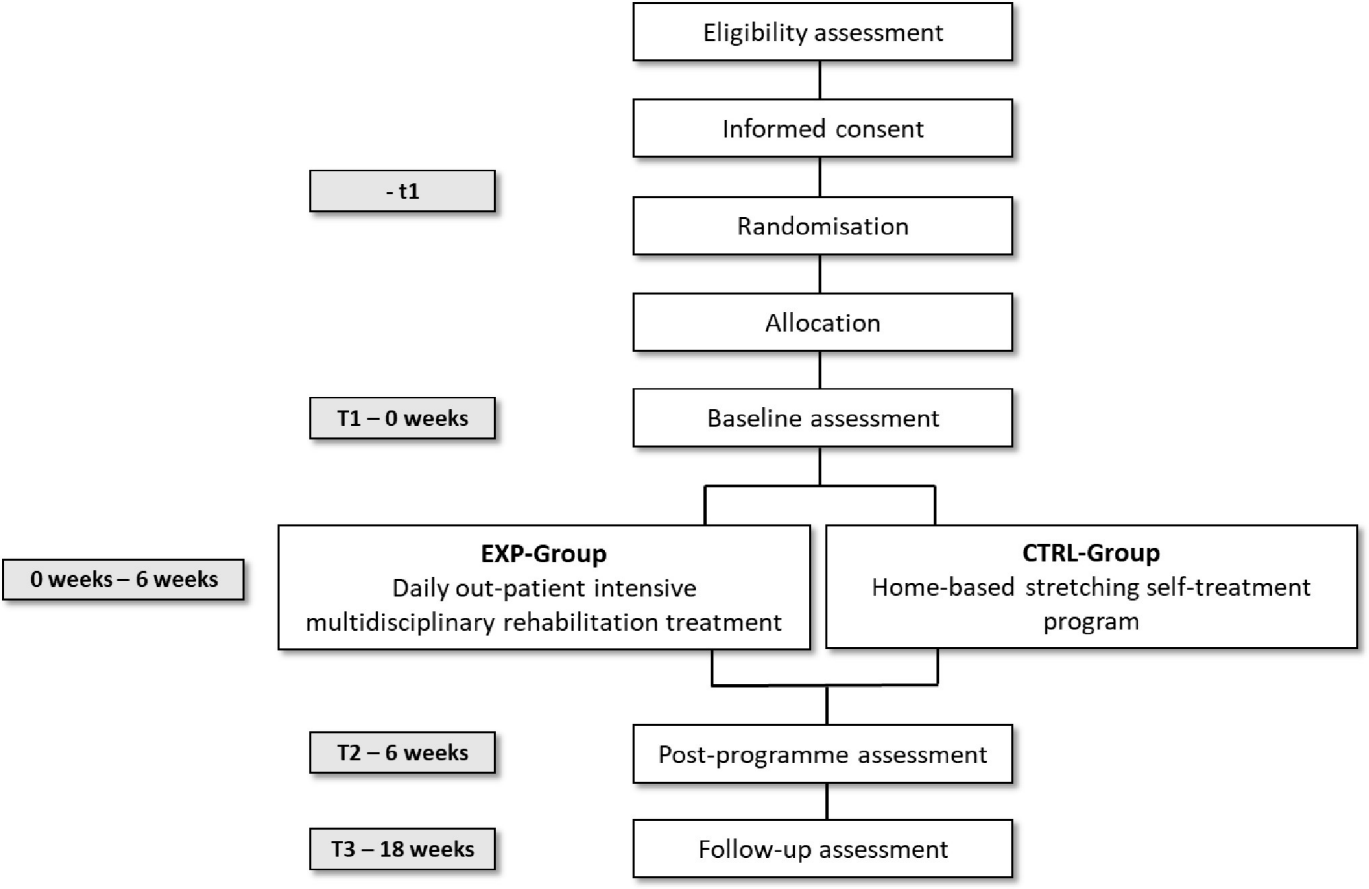
Participant timeline (time schedule of enrolment, interventions, and assessments).

Participants conforming to the inclusion criteria are randomly allocated to the EXP-Group or to the CTRL-Group. Each participant then undergoes multidimensional in-depth assessment before (T1), at the end of treatment (T2) and 3 months after the end of treatment (T3). Fig 2 outlines the participant timeline (time schedule of enrolment, interventions and assessments).

### Study setting

The study takes place at the Center for Diagnosis and Rehabilitation of Parkinson’s Disease and Parkinsonisms (DiaRiaPARK) of the IRCCS Fondazione Don Carlo Gnocchi ONLUS in Milan. PwPD allocated in EXP-Group carry out the rehabilitation program within the setting called Complex Outpatient Macroactivity (i.e. Macroattività Ambulatoriale Complessa, “MAC”) while the participants allocated to the control group carry out the self-treatment program at home. All assessments for each time point are performed in the clinic.

### Subject recruitment

Participants are consecutively recruited at the DiaRiaPARK. Recruitment of participants started on December 9, 2020, and is anticipated to be completed by December 31, 2024. Once the subjects are selected, they receive a detailed explanation of the research procedures, and the subjects voluntarily express their consent to participate in this study, signing the informed consent.

### Sample size

There are currently no clinical studies examining the effectiveness of rehabilitation treatment on our primary outcomes. Therefore, the sample size was determined based on the preliminary results of a recent cross-sectional study by Agliardi and colleagues [36]. In Agliardi’s study a total of 32 PD patients were consecutively recruited and screened and a total of 40 healthy subjects were included as controls using frequency matching for age and gender. The main objective of this study was to identify differences in NDEs derived oligomeric α-Synuclein concentration between PD and healthy controls subjects. Hence, considering 2 degrees of freedom with 80% power; 0,05 alpha and a drop-out percentage of 20%, we planned a sample size of N = 72 (N for each group = 36) that will be more than adequate to control for a potential participants’ drop out.

### Eligibility criteria

#### Inclusion criteria

Clinical diagnosis of PD by a movement disorder specialist according to MDS Criteria [37], participants aged between 50 and 85 years, modified Hoehn & Yahr (mH&Y) [38] stage from 1.5 to 3 and stable pharmacological treatment in the previous 4 weeks.

#### Exclusion criteria

Vascular, familiar and drug- induced forms of parkinsonism, other known or suspected causes of parkinsonism (e.g., metabolic, brain tumour etc) or any suggestive features of atypical parkinsonism; significant comorbidities and/or severe systemic diseases that would preclude exercise participation (e.g. recent surgery, unstable cardiac dysfunction, anemia, hepatosis, pulmonary disorders, chronic renal failure; auditory, visual and/or vestibular dysfunctions, presence of DBS); previously diagnosed psychiatric diseases; clinical diagnosis of dementia as defined by Montreal Cognitive Assessment (MoCA Test) Correct Score<15.51 [39]; rehabilitation treatment in the previous 4 weeks. If the participant is unable to comply with inclusion criteria or currently participating in another interfering research project or undergoing any interfering therapy, he/she is not be recruited.

### Randomization and blinding

Once recruited, participants are randomly allocated to two different groups, the Experimental (EXP-Group = 36) and the Control Group (CTRL-Group = 36). A sample block randomization method is employed. The random allocation table is generated by computer and stratified based on PD stages according to mH&Y classification (from 1.5 to 3).

The study involves a single-blind procedure; the allocation of subjects to intervention or control groups is blinded to assessors. Participants are blinded to the benefits of each intervention and the study hypothesis.

Blinded assessments are performed at T1, T2, and T3 by a movement disorder specialist.

### Participant engagement

The study’s details and timescales are explicitly discussed with participants before starting the treatment to ensure they are fully aware of the demands of it, as well as the potential impact on fatigue levels. In addition, the study has the added benefit of providing participants with the opportunity to receive a comprehensive evaluation of their clinical status (i.e., neurological, motor, and neuropsychological evaluation), which is rarely offered, as part of routine care.

### Interventions

#### Experimental group

The rehabilitation program lasts 6 consecutive weeks and involves the execution of a total of 30 days, one for each day of the week (from Monday to Friday) in an outpatient setting. Sessions last 160’/day (80’ motor, 40’ cognitive, and 40’ speech therapy rehabilitation) for 3 days a week and 180’/day (80’ motor, 60’ cognitive, and 40’ speech therapy rehabilitation) for 2 days a week.

*Motor rehabilitation*. The motor rehabilitation program is based on relevant literature and clinical guidelines [40]. The total number of physical therapy sessions is 60, each session lasts 40 minutes and 2 sessions are provided per day. A “circuit” training is performed in 18 out of 60 sessions, 3 times per week on alternate days. The “circuit” training comprised the combination of aerobic training on a treadmill and task-oriented balance exercises in the same session, as previously shown in the study conducted by Soke et al. [41]. During the aerobic training, participants walk on a treadmill for 20 minutes while maintaining a Rate of Perceived Exertion (RPE) between 13 and 15 points (moderate-high intensity of the activity). The RPE can be considered as a proxy of a physiological parameter, assessing the intensity of physical activity perceived during the activity itself [42]. The speed and slope gradually increase and adjust to achieve the desired RPE level. In the same session, the remaining 20 minutes are dedicated to improving static and dynamic stability using a task-oriented approach that promotes explicit motor relearning. The approach involves information on the relative position of the center of mass with respect to the base of support, controlling axial segments, and using vestibular, visual, or proprioceptive information based on the subject’s specific impairments.

Due to the high effort required for the “circuit” training, the physical therapist has the freedom to choose exercises for the participants in the remaining 42 sessions of “conventional” training (7 x week). These exercises will be based on the clinical needs of the individuals, following the recommendations provided in the guidelines [40]. Some examples of exercises that may be included are resistance training, dual-task exercises, biofeedback exercises, joint mobilization, and exercises to manage the freezing of gait.

*Cognitive rehabilitation*. Cognitive rehabilitation or enhancement treatment involves an individualized program developed and supervised by a neuropsychologist; such treatment is mainly targeted frontal/executive/attentive/visuo-spatial-constructive functions in addition to memory and language functions. The cognitive treatment is proposed both in traditional mode (40’; 3 times/week) and through the support of a semi-immersive "Virtual Reality Rehabilitation System” (VRRS) [43] (60’; 2 times/week). The VRRS protocol consists of individual cognitive exercises for 6 weeks, 2 times a week for 60 minutes per session (N = 12). Each session is planned with six cognitive exercises lasting 10 minutes, designed to improve executive functions, visuospatial abilities, attention, and memory. A pre- and post- training sessions are conducted to tailor the cognitive intervention to the subjects’ baseline performance and to evaluate the abilities at the end of treatment. At the end of each session, subjects receive feedback on their performances, and a detailed report of the results is available to the therapist, allowing the monitoring of progress over time. Progression is constantly monitored, and the exercises adaptively modulated in difficulty.

*Speech therapy*. Participants undergo an individualized program supervised by a speech therapist that includes assessment and treatment of voice (dysphonia), articulation (dysarthria), and deglutition (dysphagia). Innovative techniques are used for both assessment (acoustic speech analysis) and treatment (biofeedback with Vitalstim) (40’; 5 times/week). In addition, recommendations for proper deglutition are provided, and counselling sessions (e.g., training about the use of ad hoc products for dysphagia) are offered to the patient and caregiver.

#### Control group

Participants allocated to the control group (CTRL-Group) carry out a 40’/day home-based self-treatment program consisting of muscle-stretching and active mobilization exercises for 40’/day for 6 consecutive weeks, from Monday to Friday. Participants receive detailed exercises instructions and a diary where exercises performed, any side effects and/or specific difficulties ran into the program should be noted. One training session will be given to the participants allocated in the control group in order to learn how to perform home exercises.

### Outcomes and measurement

The primary outcome of this study is changes in molecular biomarkers levels (blood biomarkers and serum NDEVs derived biomarkers) in response to an intensive multidisciplinary rehabilitation treatment in PwPD (see Table 1). Secondary outcomes include changes in motor and non-motor symptoms, balance and gait performance, aerobic capacity, and cognitive functioning (see Table 2).

**Table 1:** Primary outcomes and instruments to assess the study variable.

Primary Outcome measures	Instruments to assess the study variable
**Blood Biomarkers**	**Serum biomarkers in NDEVs** Oligomeric α-synuclein (α-syn) ng/ml; SNARE complex: Syntaxyn-1(STX-1A) (ng/ml), VAMP-2 (ng/ml) and SNAP-25 (ng/ml); Brain-Derived Neurotrophic Factor (BDNF) (ng/ml); Pro-BDNF (ng/ml); Glial cell line-derived Neurotrophic factor (GDNF) (ng/ml); Cerebral dopamine neurotrophic factor (CDNF) (ng/ml). **Pro-inflammatory** [IL-1β (pg/ml), Tumour Necrosis Factor alpha (TNFα) (pg/ml), Interferon gamma (IFN-γ) (pg/ml), IL-6 (pg/ml), IL-18 (pg/ml)], Anti-inflammatory (IL-10) (pg/ml) cytokines.

**Table 2:** Secondary outcomes and instruments to assess the study variable.

Secondary Outcome measures	Instruments to assess the study variable
**Functional assessment and motor function**	• The Movement Disorders Society Unified Parkinson’s Disease Rating Scale (MDS-UPDRS) Part I-IV • Timed-Up and Go test (TUG) • 6-Minute Walk Test (6-MWT) • 10-Meter Walk Test (10MWT) • 5-Time Sit-To-Stand (5TSTS) • Modified Dynamic Gait Index (mDGI) • Gait analysis (using a 9-camera SMART-D motion capture system (Optional)
**Cognitive functioning**	• Montreal Cognitive Assessment (MoCA Test) • Mini-Mental Parkinson (MMP) • Forward Verbal Span • Backward Verbal Span • Immediate and delayed story recall test • Rey’s Figure–Recall • Rey-Osterrieth Complex Figure Copying Test • Frontal Assessment Battery (FAB) • Raven Coloured Progressive Matrices (CPM-47) • Attentive Matrices • Alternate Verbal Fluency • Verbal fluency test (phonemic and semantic tasks) • Trail Making Test (TMT) • Symbol Digit Modalities Test (SDMT) (Oral Version) • Stroop Test-Short Version • Boston Naming Test • Gesture Imitation Test (IMA-T)
**Non-motor symptoms**	• Non-Motor Symptoms Scale (NMSS) • Parkinson Fatigue Scale (PFS) • Epworth Sleepiness Scale (ESS) • Pittsburgh Sleep Quality Index (PSQI) • REM sleep behavior disorder screening questionnaire (RBDSQ) • Italian version of the Composite Autonomic Symptoms Score (COMPASS-31) • Numeric Rating Scale (NRS) • The Parkinson Disease Questionnaire (PDQ-39) • Beck Depression Inventory-II (BDI-II) • State-Trait Anxiety Inventory. Forma Y (STAI-Y) • Dimensional Apathy Scale (I-DAS) • Snaith-Hamilton Pleasure Scale (SHAPS) • Barratt Impulsiveness Scale-11 (BIS-11) • Toronto Alexithymia Scale (TAS-20) • Questionnaire for Impulsive-Compulsive Disorders in Parkinson’s disease (QUIP-RS-IT) • NeuroPsychiatric Inventory Questionnaire (NPI-Q)
**Daily self-care activities**	• Activities of Daily Living (ADL) • Instrumental Activities of Daily Living (IADL)

Each participant undergoes assessments before (T1), at the end of treatment (T2) and 3 months after the end of treatment (T3). The measures use for these assessments, applied at each timepoint, are detailed in Fig 1.

At the baseline a researcher collects socio-demographic data from each participant (i.e., age, gender, education level and hand dominance). Then, blood sample collection and assessment of disease severity, cognition, motor and non-motor symptoms are performed at T1, T2 and, T3.

### Data management

Signature of informed consent, data collection, and the experimental treatment are conducted in person.

Each patient is given an identification code to guarantee their anonymity in the study. Separate storage is made for a list of identification codes. Descriptive data are gathered and stored in data files on protected computer networks.

Blood samples are delivered at the Laboratory of Molecular Medicine and Biotechnology (LAMMB) of the IRCCS Fondazione Don Carlo Gnocchi ONLUS—S. Maria Nascente in Milan where they are marked with an anonymous code, and then processed for serum storage and NDEVs enrichment (See S1 and S2 Appendices). All anamnestic, clinical, and biological data are collected in an anonymized database with a progressive number code and analyzed as aggregate data. Signed informed consent and all clinical documents are stored in a guarded archive in the PI office.

### Monitoring

The research team has expertise in a variety of disciplines, such as medical and behavioral management, patient engagement, biostatistics, quantitative and qualitative outcomes assessment and analysis.

The local ethics committee does not require a data monitoring committee due to the short duration and minimal risks involved in this rehabilitation program.

The team records any adverse events that participants report, assesses them internally to prevent future ones, and reports them as part of the planned publication.

Since there are no sponsors for the study, it is not necessary to provide information on the frequency and procedures for reviewing the study process. Weekly meetings are held by the project management group to discuss the current status of data collection and address potential problems and difficulties.

### Data analysis

The analyses will be conducted using SAS software version 9.4 and R software version 4.2.1. We will conduct a descriptive analysis of the two treatment groups, including the mean and standard deviation for normally distributed variables and the median and interquartile range for variables deviating significantly from normality. To assess the treatment effect, we will employ an analysis of covariance (ANCOVA) model [44], incorporating covariates (gender, age, and disease duration) to adjust the estimates. This model will be utilized to evaluate the treatment effect both at the conclusion of the treatment period and at the end of follow-up time. The parametric approach will be applied for primary outcomes, characterized by continuous scales. Concerning secondary outcomes, non-parametric tests (Wilcoxon rank-sum test) will be employed for those assessed with ordinal scales. Estimates of primary outcomes will be adjusted using False Discovery Rate correction [45]. A 95% confidence interval will be employed, and statistical significance will be reported for any observed differences between groups with a significance level set at p < 0.05. Subgroup analyses will be conducted to examine whether the treatment exhibits a differential effect based on the severity of the patient, as determined by baseline values of the Hoehn and Yahr scale or UPDRS III scale.

### Ethical approval and registration

The design of this study conforms to the principles outlined in the Declaration of Helsinki and was approved by the “Fondazione Don Carlo Gnocchi-Milan” Ethics Committee, project identification code 1_16/04/2020, and subsequent amendments identified with ID 8/2021/CE_FdG/FC/SA and with ID 13/2023/CE_FdG/FC/SA. The examiner provides all participants with a complete explanation of the purpose and risk of the study before they sign a written informed consent based on the revised Declaration of Helsinki (2013). Participation in the study is voluntary and requires written informed consent from each participant. Eligible patients are informed about all relevant aspects of this study before commencing the study. This RCT was registered on May 30, 2022, to ClinicalTrials.gov with the Study ID number: NCT05452655.

### Trial status

As of the time of this publication, the study is actively underway in accordance with protocol n. 13/2023/CE_FdG/FC/SA, dated April 14, 2023. Recruitment of participants started in December 9, 2020, and is anticipated to be completed by December 31, 2024.

## Discussion

Scientific literature is increasingly revealing that integrated motor and cognitive aerobic rehabilitation approaches are effective in improving motor and non-motor symptoms in PD. The effects of this type of rehabilitation on the biomolecular mechanisms of PD pathology is still being evaluated by a limited number of studies.

We assume that this rehabilitation program can be crucial in modifying biomolecular and functional parameters in PwPD. We expect that this study will bring further evidence to understanding the impact of an aerobic and intensive rehabilitation program on brain plasticity in PD. Moreover, the current project’s results will give more details about the effectiveness on motor and non-motor symptoms of an intensive multidisciplinary rehabilitation treatment for people living with PD.

We believe that there could be some limitations in the study. Given the great effort required from patients, we may encounter difficulties in recruiting participants and dropouts may occur. However, considering the number of dropouts reported in recent works from Ferrazzoli et al. (2018) [14] and Frazzitta et al. (2015) [16], we assume that the treatment we propose can be tolerated and have a minimal dropout rate. The single-blind design of this study could be another limitation due to the possibility of the placebo effect. In that regard, due to several factors (e.g., patients’ heterogeneity, blinding difficulties and proper controlled condition), the process of implementing double-blind randomized placebo controlled trials can be very difficult in an inpatient rehabilitation setting.

## Supporting information

S1 Checklist SPIRIT checklist. (DOC)

S1 Appendix Informed consent materials. (PDF)

S2 Appendix Biological specimens. (DOCX)

S1 Protocol Clinical trial protocol_It. Clinical trial protocol_Italian Version (Ethics approved).
(PDF)

S2 Protocol Clinical trial protocol_En. Clinical trial protocol_English Version (Ethics approved).
(PDF)
