# Supplementary material for: Investigating the effects of a daily multidisciplinary intensive outpatient rehabilitation program on innovative biomarkers in people with Parkinson’s disease: Study protocol for a phase III randomized controlled clinical trial
Source: PLoS One. 2024 Oct 23;19(10):e0309405. doi: 10.1371/journal.pone.0309405 (PMC11498734; doi:10.1371/journal.pone.0309405)
Supplement: S1 Appendix — (PDF) [file pone.0309405.s002.pdf]

FOGLIO INFORMATIVO E MODULO DI CONSENSO INFORMATO ALLA PARTECIPAZIONE AL PROGETTO DI RICERCA

Studio Interventistico Malattia di Parkinson

Titolo dello studio:

"CARATTERIZZAZIONE DELLE VESCICOLE EXTRACELLULARI DI ORIGINE NEURALE E GLIALE PER L'INDIVIDUAZIONE DI AFFIDABILI BIOMARKERS MOLECOLARI PER LA MALATTIA DI PARKINSON (MP), PER I PARKINSONISMI ATIPICI E PER IL DISTURBO DEL COMPORTAMENTO NELLA FASE REM IDIOPATICO."

Egr. Sig. / Gent.le Sig.ra

La informiamo che stiamo conducendo presso il Laboratorio di Medicina Molecolare e Biotecnologie dell'IRCCS S. Maria Nascente, Milano, della Fondazione Don Carlo Gnocchi, uno studio dal titolo: "*Caratterizzazione delle vescicole extracellulari di origine neurale e gliale per l'individuazione di affidabili biomarkers molecolari per la Malattia di Parkinson (MP), per i Parkinsonismi atipici e per il Disturbo del comportamento nella fase REM idiopatico*."

Per realizzare questo studio desideriamo avvalerci della collaborazione e della disponibilità di persone come Lei che soddisfino i requisiti scientifici idonei all'inserimento nel gruppo di soggetti affetti da malattia di Parkinson, per questo motivo Le proponiamo di partecipare al presente studio che sarà condotto sotto la responsabilità scientifica della Dr.ssa Francesca Lea Saibene.

Con il presente documento chiediamo la Sua autorizzazione alla partecipazione allo studio.

Prima che Lei decida, La invitiamo a leggere con attenzione questo documento, qualora Lei desideri avere ulteriori informazioni e chiarimenti potrà rivolgersi al/alla Dr./Dr.ssa ______________________________ (i cui recapiti sono indicati in fondo al presente documento) che Le dedicherà tutto il tempo necessario per chiarire ogni Suo dubbio, potrà contattarci ai recapiti indicati al termine del foglio informativo.

Premesse e scopo dello studio

Una sfida importante nello studio delle malattie Neurodegenerative, quali la malattia di Parkinson, è quella di individuare nuovi biomarcatori per inquadrare meglio e monitorare l'evoluzione della malattia in quanto quelli attualmente disponibili sono di utilità limitata e poco adeguati per le proiezioni su larga scala.

Con il nostro studio vogliamo approfondire il ruolo delle proteine tossiche responsabili dell'esordio e della progressione di questa patologia.

Perché vogliamo svolgere questo studio

Le patologie Neurodegenerative sono caratterizzate da una forte variabilità di manifestazione e di decorso clinico, oltre che di declino cognitivo, funzionale e comportamentale. La progressione di malattia dipende fortemente dalla comunicazione delle cellule nervose (plasticità neuronale) nei tessuti cerebrali. La progressiva e costante perdita di neuroni caratterizza la malattia di Parkinson, questa neurodegenerazione provoca riduzione dei livelli di dopamina, compromettendo alcune funzioni tra le quali il controllo dei movimenti e dell'equilibrio. La perdita dei neuroni dopaminergici inizia almeno 20 anni prima del manifestarsi dei primi sintomi, questa fase è detta prodromica. Per questo

motivo è di urgente interesse individuare biomarcatori di malattia precoci, specifici e facilmente accessibili. La morte dei neuroni è causata dall'accumulo di una proteina tossica per le cellule che si chiama α-sinucleina (α-Syn). Non sono ancora noti i processi attraverso cui l'α-Syn inizia ad aggregare e causare neurotossicità. L'α-Syn è disponibile fisiologicamente nelle cellule dove interagisce con altre proteine (il complesso proteico SNARE) per il corretto funzionamento delle sinapsi neuronali e il rilascio dei neurotrasmettitori. Con il termine vescicole extracellulari (EVs) si intendono esosomi e microvescicole, nanoparticelle che si differenziano sia per la dimensione, sia per la modalità con cui originano dalla cellula. Le EVs partecipano alla comunicazione tra le cellule trasportando proteine e acidi nucleici dalla cellula di origine alle cellule recipienti dove esplicano la loro funzione biologica; possono essere isolate da quasi tutti i fluidi biologici quali il sangue, le urine, la saliva, il liquido seminale, il liquido amniotico, il liquido sinoviale, il liquido cefalorachidiano e il latte materno. Le EVs di origine neurale così isolate possono quindi essere studiate nel loro contenuto allo scopo di individuare biomarcatori periferici facilmente accessibili, indicatori di patologie del sistema nervoso centrale (SNC).

Il nostro gruppo ha recentemente dimostrato che alcune proteine del complesso SNARE sono presenti negli esosomi periferici di tipo neurale. La misurazione periferica di queste proteine potrebbe essere un indice di integrità sinaptica di facile accesso che rispecchia quello che avviene a livello cerebrale durante la patologia.

Pertanto il nostro progetto ha lo scopo di identificare nuovi parametri utili per definire la progressione della malattia e per indicare nuove strategie preventive, diagnostiche e terapeutiche.

Procedure previste dallo studio

Lo studio prevede la partecipazione di soggetti con diagnosi di Malattia di Parkinson afferenti presso l'ambulatorio Parkinson di I° e II° livello; Macroattività Ambulatoriale Complessa (MAC) Parkinson e l'U.O. di Neurologia Riabilitativa dell'IRCCS S. Maria Nascente, Fondazione Don C. Gnocchi, Milano.

La realizzazione dello studio richiede l'arruolamento di un gruppo di pazienti (in cui Lei sarà incluso) formato da soggetti con diagnosi di Malattia di Parkinson.

Le sarà chiesto di effettuare un prelievo di 37 ml di sangue venoso. Il campione prelevato sarà consegnato al Laboratorio di Medicina Molecolare e Biotecnologie dell'IRCCS, dove sarà di prassi contrassegnato con codice anonimo, e poi processato per: stoccaggio di siero/plasma, estrazione di DNA e miRNA. Su tali campioni verranno caratterizzati molecolarmente i polimorfismi dei geni ApoE, SNAP-25, STX1a e VAMP2 e verrà testato un pattern di miRNA correlati al gene SNAP-25.

Tutti i dati anamnestici, clinici e biologici verranno raccolti in un database anonimizzato tramite codice numerico progressivo e valutati come dati aggregati.

Gli elementi ottenuti dal campione di sangue saranno conservati alla temperatura di -80°C presso il suddetto Laboratorio sotto la responsabilità del Direttore (Prof. Mario Clerici). Il campione di sangue sarà poi analizzato presso il Laboratorio per le indagini di tipo biologico previste dallo studio.

Nel caso in cui Lei si ritiri dallo studio, dopo l'effettuazione del prelievo di sangue, il campione acquisito sarà immediatamente distrutto e non sarà effettuata nessuna ulteriore valutazione sul campione stesso, salvo che, in origine o a seguito del trattamento, dal campione non sia più possibile risalire alla Sua identità.

Il campione di materiale biologico prelevato sarà utilizzato per lo scopo del presente studio senza alcun fine di lucro, qualora vi sarà la possibilità di utilizzare il campione nell'ambito di progetti i cui

scopi e le cui metodologie di ricerca non risultino conformi a quelli descritti nel presente documento, Le sarà chiesto di rilasciare per iscritto un ulteriore nuovo e specifico consenso.

Nel caso in cui Lei decida di partecipare allo studio verrà arruolato e allocato in modo casuale o per essere sottoposto ad un trattamento riabilitativo presso la nostra clinica oppure per essere incluso in un programma di auto-trattamento domiciliare; sarà quindi sottoposto alle valutazioni cliniche e al prelievo di sangue all'inizio, alla fine del trattamento e dopo 3 mesi dalla fine del trattamento.

Il trattamento riabilitativo presso il nostro Istituto durerà 6 settimane e prevedrà l'esecuzione di 30 sedute, 5 giorni a settimana della durata di 160 minuti ciascuna (80 minuti di riabilitazione motoria; 40 minuti di riabilitazione cognitiva e 40 minuti di riabilitazione logopedica) per 3 giorni alla settimana e 180 minuti (80 minuti di riabilitazione motoria; 60 minuti di riabilitazione cognitiva e 40 minuti di riabilitazione logopedica) per 2 giorni alla settimana. All'interno dell'intervento di riabilitazione motoria saranno proposte 18 sedute (3 volte alla settimana) in cui è previsto un lavoro aerobico (20 minuti) seguiti da esercizi di equilibrio e rinforzo funzionale (20 minuti).

Le rimanenti sedute di riabilitazione motoria saranno di tipo convenzionale comprendenti esercizi definiti dal terapista secondo le sue necessità terapeutiche.

Il trattamento di riabilitazione o potenziamento cognitivo sarà supervisionato da un neuropsicologo. Tale trattamento sarà rivolto principalmente alle funzioni cognitive e sarà proposto sia in modalità tradizionale (3 volte/settimana) sia attraverso il coinvolgimento in attività condotte con il supporto della realtà virtuale semi-immersiva (2 volte/settimana).

È inoltre previsto un programma riabilitativo supervisionato da un logopedista che comprenderà la valutazione ed il trattamento della voce (disfonia), dell'articolazione (disartria) e della deglutizione (disfagia).

Il trattamento riabilitativo presso il proprio domicilio durerà altrettante 6 settimane e consiste in un programma di auto-trattamento della durata di 40 minuti al giorno per 5 giorni a settimana. Il programma riabilitativo consiste in esercizi di stretching e di mobilizzazione attiva.

Prima di iniziare il programma Lei assisterà ad una seduta dimostrativa con il fisioterapista e Le verranno consegnate le istruzioni dettagliate degli esercizi e un diario dove dovrà annotare gli esercizi effettuati, gli eventuali effetti collaterali e/o le difficoltà specifiche incontrate durante il programma. L'aderenza al trattamento verrà monitorata una volta a settimana tramite contatto telefonico.

In fase di arruolamento, sarà chiesta la disponibilità al monitoraggio delle attività motorie al domicilio attraverso l'indosso di nuovi strumenti di monitoraggio. Le sarà chiesto di indossare per una settimana un sensore assimilabile a un orologio da polso a ciascun polso. L'orologio potrà essere tolto per necessità o fastidio e indossato successivamente. Le misure effettuate dai sensori permettono di quantificare il livello di attività maggiore o minore; ma non permettono di identificare quale attività si sta svolgendo in un determinato momento.

Il principale obiettivo consiste nel raccogliere informazioni riguardanti le caratteristiche dei movimenti eseguiti in ambiente domestico. Durante questa settimana è inoltre richiesto di annotare su un Diario (che Le sarà fornito) le principali attività motorie svolte durante la giornata e l'assunzione della terapia farmacologica. Dopo una settimana, entrambi i dispositivi saranno rimossi e le informazioni registrate saranno dapprima scaricate su un computer, quindi analizzate con un algoritmo creato ad hoc da un bioingegnere. La stessa procedura sarà quindi svolta alla fine del trattamento riabilitativo e dopo 3 mesi dalla fine del trattamento riabilitativo.

Partecipazione allo studio

La partecipazione è completamente libera e volontaria.

Se Lei acconsente a partecipare Le sarà chiesto di firmare il Modulo di Consenso Informato, allegato al presente documento, prima che Lei inizi a eseguire la procedura prevista dallo studio.

La firma del modulo allegato è al fine di garantire che Lei abbia ricevuto un'informazione completa e che abbia espresso liberamente la Sua volontà di partecipare; tale firma non implica alcun impegno da parte Sua a proseguire lo studio, non costituisce un vincolo di natura contrattuale, né rappresenta una rinuncia ai diritti che Le spettano.

Nel caso in cui Lei decida di ritirarsi dallo studio, dopo avere inizialmente accettato, potrà interrompere la Sua partecipazione in qualsiasi momento dandone comunicazione al responsabile dello studio senza dover fornire una giustificazione. La scelta di non partecipare, o di ritirarsi dopo l'iniziale accettazione, non comporta l'esclusione o la limitazione delle cure e dell'assistenza che Lei riceve presso le nostre strutture, né alcuna penalizzazione nel Suo rapporto con il personale che La assiste.

Qualora si venisse a conoscenza di nuovi dati o di risultati che possano influenzare la Sua partecipazione allo studio ne sarà tempestivamente informato/a; inoltre, il Responsabile dello studio potrà ritirarLa dallo studio qualora ritenga che tale decisione risponda al Suo migliore interesse.

Sul piano economico la partecipazione allo studio non determina alcun tipo di onere o di spesa aggiuntiva a Suo carico.

Precisiamo che non Le viene richiesto di partecipare a questo studio per ricevere assistenza clinica, o per ottenere beneficio personale di tipo diagnostico.

Possibili benefici relativi alla partecipazione allo studio.

La Sua partecipazione allo studio permetterà di acquisire informazioni e di sviluppare conoscenze che saranno di utilità sia per Lei che per altri soggetti.

Possibili rischi / effetti collaterali legati alla partecipazione allo studio.

La partecipazione a questo studio non Le comporterà alcun rischio o disagio in quanto non determina variazioni nella gestione del trattamento ordinario, né implica la necessità di eseguire procedure o interventi aggiuntivi. Le chiediamo la disponibilità a sottoporsi ad un prelievo di sangue che sarà eseguito secondo la comune procedura in uso presso tutti i laboratori di analisi. Al termine del prelievo potrebbe manifestarsi un piccolo ematoma o un livido nella zona del braccio in cui il sangue è stato prelevato, per evitare tale inconveniente è consigliabile, alla fine del prelievo, esercitare una lieve pressione sul punto dove è avvenuta la puntura. Particolare attenzione, durante il prelievo, verrà posta ai soggetti sottoposti a terapia con anti-aggreganti.

Il Protocollo dello studio a cui Le viene proposto di partecipare, è stato approvato, unitamente al presente documento, dal Comitato Etico della Fondazione Don Carlo Gnocchi.

Per ulteriori informazioni, chiarimenti e comunicazioni:

Dr.ssa Francesca Lea Saibene

U.O. Neurologia Riabilitativa, IRCCS S. Maria Nascente

Tel.: 02-40.30.88.33

E-mail: fsaibene@dongnocchi.it

Dr. Jorge Navarro

U.O. Neurologia Riabilitativa, IRCCS S. Maria Nascente

E-mail: jnavarro@dongnocchi.it

Prof. Mario Clerici

Laboratorio di Medicina Molecolare e Biotecnologie, IRCCS S. Maria Nascente

Tel.: 02-40.30.83.76

E-mail: mclerici@dongnocchi.it

Dr.ssa Franca Guerini

Laboratorio di Medicina Molecolare e Biotecnologie, IRCCS S. Maria Nascente,

Fondazione Don C. Gnocchi ONLUS,

Tel.: 02-40308211

E-mail: fguerini@dongnocchi.it

Dr.ssa Cristina Agliardi

Laboratorio di Medicina Molecolare e Biotecnologie, IRCCS S. Maria Nascente,

Fondazione Don C. Gnocchi ONLUS,

Tel.: 02-40308211

E-mail: cagliardi@dongnocchi.it

La ringraziamo per la Sua disponibilità e la Sua collaborazione

MODULO DI CONSENSO INFORMATO per la partecipazione allo studio:

Studio Interventistico Malattia di Parkinson

Titolo dello studio:

"CARATTERIZZAZIONE DELLE VESCICOLE EXTRACELLULARI DI ORIGINE NEURALE E GLIALE PER L'INDIVIDUAZIONE DI AFFIDABILI BIOMARKERS MOLECOLARI PER LA MALATTIA DI PARKINSON (MP), PER I PARKINSONISMI ATIPICI E PER IL DISTURBO DEL COMPORTAMENTO NELLA FASE REM IDIOPATICO".

Io sottoscritto/a: ____

Cognome e Nome in stampatello del soggetto adulto partecipante.

nato/a a, il: ____

Luogo e data di nascita del soggetto adulto partecipante.

DICHIARO QUANTO SEGUE:

1. ho letto e compreso il foglio informativo di cui questo modulo è parte integrante;
2. ho avuto la possibilità di porre domande e di chiedere spiegazioni al/alla Dr./Dr.ssa ______________________________ dal/dalla quale ho ricevuto risposte soddisfacenti;
3. mi sono state illustrate la natura, lo scopo e la durata dello studio, le procedure che saranno seguite, il trattamento previsto per i partecipanti e il tipo di collaborazione che ad essi sarà richiesta;
4. ho compreso che la mia partecipazione è libera e volontaria e che in qualsiasi momento posso decidere di interrompere la mia partecipazione e ritirarmi dalle ricerche senza dovere fornire giustificazione e senza essere in alcun modo privato/a delle cure e dell'assistenza di cui ho bisogno e dell'eventuale accesso a nuove prospettive diagnostiche e/o terapeutiche e senza che siano compromessi i miei diritti e il mio rapporto con il medico e con gli operatori sanitari.

Tutto ciò premesso, nella mia piena capacità di intendere e di volere e senza alcuna forma di condizionamento o coercizione, accetto la proposta di partecipare allo studio descritto nel presente documento.

Luogo e data: ________________________ Firma: ________________________

FIRMA DEL TESTIMONE (ove applicabile)

Se la persona partecipante allo studio, non è in grado di leggere o di firmare, un testimone indipendente dal promotore e dallo sperimentatore, deve firmare e datare personalmente la dichiarazione di consenso informato dopo che l'informativa sia stata letta e spiegata al soggetto e questi abbia manifestato il proprio inequivocabile consenso a partecipare allo studio.

Cognome e Nome in stampatello del testimone: ____

Luogo e data: ______________________ Firma: _______________________

PARTE RISERVATA ALL'OPERATORE CHE HA PRESENTATO L'INFORMATIVA

Io sottoscritto/a Dr/Dr.ssa __________________________ *(Cognome e Nome in stampatello)*

DICHIARO:

a. di avere spiegato alla persona sopraindicata la natura e lo scopo dello studio, nonché le procedure che saranno adottate e il tipo di collaborazione che sarà richiesta;

b. di non avere cercato di influenzare o di costringere in alcun modo la persona sopra indicata per indurla a manifestare il suo consenso alla partecipazione allo studio;

c. di rilasciare alla persona sopraindicata una copia firmata e datata del presente modulo insieme al foglio informativo.

Luogo e data: ______________________ Firma: _______________________

MODULO DI CONSENSO INFORMATO per:

PRELIEVO, CONSERVAZIONE E UTILIZZO DEL MATERIALE BIOLOGICO PER FINALITÀ DI RICERCA

Studio Interventistico Malattia di Parkinson

Titolo dello studio:

"CARATTERIZZAZIONE DELLE VESCICOLE EXTRACELLULARI DI ORIGINE NEURALE E GLIALE PER L'INDIVIDUAZIONE DI AFFIDABILI BIOMARKERS MOLECOLARI PER LA MALATTIA DI PARKINSON (MP), PER I PARKINSONISMI ATIPICI E PER IL DISTURBO DEL COMPORTAMENTO NELLA FASE REM IDIOPATICO".

Io sottoscritto/a: ____

Cognome e Nome in stampatello del soggetto adulto partecipante.

nato/a a, il: ____

Luogo e data di nascita del soggetto adulto partecipante.

dopo essere stato/a informato/a che:

1. presso il Laboratorio di Medicina Molecolare e Biotecnologie dell'IRCCS S. Maria Nascente della Fondazione don Carlo Gnocchi è in corso il seguente studio: ***"Caratterizzazione delle vescicole extracellulari di origine neurale e gliale per l'individuazione di affidabili biomarkers molecolari per la Malattia di Parkinson (MP), per i Parkinsonismi atipici e per il Disturbo del comportamento nella fase REM idiopatico."*** che coinvolge pazienti affetti da malattia di Parkinson e che richiede per la realizzazione il prelievo dai partecipanti di un campione di sangue venoso (circa 37 ml);
2. il materiale biologico prelevato verrà utilizzato dal personale del Laboratorio di Medicina Molecolare e Biotecnologie esclusivamente per le finalità di ricerca connesse all'esecuzione dello studio, senza alcun fine di lucro e previa approvazione del suddetto studio del Comitato Etico della Fondazione;
3. il materiale biologico prelevato verrà conservato in condizioni di sicurezza e secondo procedure scientificamente riconosciute come indicato nell'apposito paragrafo del foglio informativo;
4. qualora vi fosse l'eventuale possibilità di utilizzare - sia presso la Fondazione Don Gnocchi sia, eccezionalmente, presso altre strutture - il materiale biologico prelevato nell'ambito di progetti i cui scopi e le cui metodologie di ricerca non risultino conformi a quelli descritti nel foglio informativo del presente studio, mi sarà chiesto di rilasciare per iscritto un ulteriore specifico consenso;
5. il materiale biologico verrà reso anonimo in modo che non sia possibile, se non al responsabile dello studio, risalire alle mie generalità;
6. avrò il diritto, nel caso in cui lo richieda, di conoscere i risultati dello studio;
7. verrà garantita la tutela della riservatezza dei dati personali in conformità con le disposizioni vigenti e con quanto precedentemente indicato nel relativo paragrafo del foglio informativo;
8. il presente consenso potrà essere in ogni momento revocato, in tal caso il campione di materiale biologico prelevato, se ancora conservato, sarà immediatamente distrutto, salvo che, in origine o a seguito del trattamento, dal campione non sia più possibile risalire alla mia identità;

DICHIARO:

➢ di essere stato/a chiaramente ed esaurientemente <u>informato/a</u> sulle attività di studio proposte e sulle esigenze che rendono indispensabile il prelievo, l'utilizzo e la conservazione del materiale biologico per le finalità dello studio e per il tempo strettamente necessario alla sua esecuzione;

➢ di prestare il consenso ☐ di non prestare il consenso ☐ *[barrare l'opzione che interessa]*

al <u>prelievo</u> del materiale biologico a me riferito e al suo trattamento esclusivamente per le finalità di ricerca scientifica legate allo studio: ***"Caratterizzazione delle vescicole extracellulari di origine neurale e gliale per l'individuazione di affidabili biomarkers molecolari per la Malattia di Parkinson (MP), per i Parkinsonismi atipici e per il Disturbo del comportamento nella fase REM idiopatico"*** e per il tempo necessario alla sua esecuzione;

➢ di autorizzare ☐ di non autorizzare ☐ *[barrare l'opzione che interessa]*

la Fondazione Don Gnocchi, IRCCS Santa Maria Nascente a <u>conservare</u> il campione biologico a me riferito presso il Laboratorio di Medicina Molecolare e Biotecnologie esclusivamente per le finalità di ricerca scientifica legate allo studio sopraindicato e per il tempo necessario alla sua esecuzione;

➢ di prestare il consenso ☐ di non prestare il consenso ☐ *[barrare l'opzione che interessa]*

alla conservazione e <u>all'ulteriore trattamento</u> del campione biologico a me riferito per lo svolgimento di studi diversi a condizione che l'utilizzo avvenga nell'ambito di progetti i cui scopi e le cui metodologie di ricerca risultino conformi a quelli descritti nel foglio informativo del presente studio e che siano stati approvati dal Comitato etico della Fondazione Don Gnocchi;

➢ di volere ☐ di non volere ☐ *[barrare l'opzione che interessa]*

essere informato/a dei <u>risultati</u> sullo studio dei campioni biologici a me riferiti, compatibilmente con i tempi necessari per la conclusione dello studio.

Luogo e Data, ____________________ Firma, _________________________

FIRMA DEL TESTIMONE (ove applicabile)

Se la persona partecipante allo studio, non è in grado di leggere o di firmare, un testimone indipendente dal promotore e dallo sperimentatore, deve firmare e datare personalmente la dichiarazione di consenso informato dopo che l'informativa sia stata letta e spiegata al soggetto e questi abbia manifestato il proprio inequivocabile consenso a partecipare allo studio.

Cognome e Nome in stampatello del testimone: ____

Luogo e data: ____________________________ Firma:______________________________

PARTE RISERVATA ALL'OPERATORE CHE HA PRESENTATO IL DOCUMENTO

Io sottoscritto/a Dr/Dr.ssa ________________________________ *(nome e cognome in stampatello)*

DICHIARO:

a. di avere informato la persona sopraindicata in merito a quanto descritto ai precedenti punti 1, 2, 3, 4, 5, 6, 7 e 8;
b. di non avere cercato di influenzarla o di costringerla in alcun modo per indurlo a dichiarare quanto sopra riportato;
c. di rilasciare alla persona sopraindicata una copia firmata e datata del presente modulo insieme al foglio informativo.

Luogo e data: ____________________________ Firma: _____________________________

Nr. Codice assegnato al soggetto partecipante: ______________________
