# Supplementary material for: Investigating the effects of a daily multidisciplinary intensive outpatient rehabilitation program on innovative biomarkers in people with Parkinson’s disease: Study protocol for a phase III randomized controlled clinical trial
Source: PLoS One. 2024 Oct 23;19(10):e0309405. doi: 10.1371/journal.pone.0309405 (PMC11498734; doi:10.1371/journal.pone.0309405)
Supplement: S2 Appendix — (DOCX) [file pone.0309405.s003.docx]

**APPENDIX**

**Biological specimens**

Blood samples are delivered at the Laboratory of Molecular Medicine and Biotechnology of the IRCCS Fondazione Don Carlo Gnocchi - S.Maria Nascente in Milan where they are marked with an anonymous code, and then processed for: serum/plasma storage, DNA extraction and miRNA. On these samples, polymorphisms of the ApoE genes, SNAP-25, STX1a and VAMP2 are molecularly characterised and tested with a miRNA pattern related to the SNAP-25 gene.  Samples were allowed to clot for 30 minutes at room temperature and then centrifuged for 10 minutes at 1500g. After centrifugation samples were aliquoted and stored at -80°C until use at the above-mentioned laboratory under the responsibility of the Director. All anamnestic, clinical and biological data are collected in an anonymised database with a progressive number code and analysed as aggregate data.
