# Supplementary material for: Investigating the effects of a daily multidisciplinary intensive outpatient rehabilitation program on innovative biomarkers in people with Parkinson’s disease: Study protocol for a phase III randomized controlled clinical trial
Source: PLoS One. 2024 Oct 23;19(10):e0309405. doi: 10.1371/journal.pone.0309405 (PMC11498734; doi:10.1371/journal.pone.0309405)
Supplement: S1 Protocol — Clinical trial protocol_Italian Version (Ethics approved). (PDF) [file pone.0309405.s004.pdf]

PROTOCOLLO DELLO STUDIO

Titolo dello studio:

"Caratterizzazione delle vescicole extracellulari di origine neurale e gliale per l'individuazione di affidabili biomarcatori molecolari per la Malattia di Parkinson (MP), per i Parkinsonismi atipici e per il Disturbo del comportamento nella fase REM idiopatico."

Responsabile dello studio

Dr.ssa Francesca Lea SAIBENE

Disegno dello studio

Studio interventistico, cross-sectional, sperimentale, controllato e randomizzato.

Tipologie di intervento

Prelievo di sangue. Il campione di sangue verrà processato presso il Laboratorio di Medicina molecolare e Biotecnologie in modo da ottenere il siero e dal siero verranno poi isolate le vescicole extracellulari (EVs) per le indagini di tipo biologico previste dallo studio.

Trattamenti riabilitativi, somministrazione di test scientificamente validati in uso nella comune prassi per la valutazione delle funzioni motorie e non-motorie, monitoraggio actigrafico per raccogliere informazioni quantitative relative all'attività motoria.

Durata prevista

Dal 01.04.2020 al 31.12.2024

Centri partecipanti allo studio e ricercatori responsabili

Gruppo di ricerca:

- Laboratorio di Medicina Molecolare e Biotecnologie, IRCCS Fondazione Don C. Gnocchi Santa Maria Nascente (Dr.ssa Cristina Agliardi: Biotecnologa ricercatrice; Dr.ssa Franca Rosa Guerini: Biologa ricercatrice; Prof. Mario Clerici: Responsabile Scientifico)

- U.O. di Neurologia Riabilitativa; Ambulatorio Parkinson di I° e II° livello; MAC Parkinson, IRCCS Fondazione Don C. Gnocchi Santa Maria Nascente (Dr.ssa Francesca Lea Saibene: Psicologa Neuropsicologa, Psicoterapeuta, Ricercatrice; Dr.ssa Anna Salvatore: Psicologa Ricercatrice; Dr. Pietro Arcuri: Medico Fisiatra; Dr.ssa Anna Castagna: Medico Neurologo; Dr.ssa Elisabetta Farina: Medico Neurologo, PhD; Dr.ssa Margherita Alberoni: Medico Neurologo; Dr.ssa Elena Calabrese: Medico Neurologo).
- LaRiCE - Laboratorio di Ricerca Cammino ed Equilibrio; IRCCS Fondazione Don C. Gnocchi Santa Maria Nascente (Dr. Davide Cattaneo: FKT, PhD; Dr. Thomas Bowman: FKT, PhD; Dr.ssa Denise Anastasi: FKT).
- Polo Tecnologico - IRCCS Fondazione Don C. Gnocchi Santa Maria Nascente (Ing. Maurizio Ferrarin: Ingegnere Biomedico; Ing. Marco Rabuffetti: Ingegnere Biomedico; Ing. Tiziana Lencioni: Ingegnere Biomedico; Ing. Ilaria Carpinella: Ingegnere Biomedico).
- CADiTeR - Centro Avanzato di Diagnostica e Terapia Riabilitativa; IRCCS Fondazione Don C. Gnocchi Santa Maria Nascente (Dr.ssa Francesca Baglio: Medico Neurologo; Dr.ssa Chiara Pagliari: Psicologa, Ricercatrice).
- U.O di Neurologia, Neurofisiologia, Neurobiologia; Dipartimento di Medicina; Università Campus Bio-Medico di Roma (Dr. Massimo Marano: Medico Neurologo)
- Fondazione IRCCS Ca' Granda Ospedale Maggiore Policlinico - Dipartimento delle Neuroscienze e Salute Mentale- Unità di Neurologia - (Dr. Alessio Di Fonzo: Medico Neurologo)
- Azienda Ospedaliero- Universitaria di Cagliari - Unità Operativa Complessa Neurologia- Centro del Sonno (Prof.ssa Monica Puligheddu: Medico Neurologo; Dr. Mario Meloni: Medico Neurologo, PhD)
- Azienda Ospedaliero-Universitaria di Padova; Dipartimento di neuroscienze-Clinica Neurologica (Prof.ssa Miryam Carecchio: Medico Neurologo; Prof. Angelo Antonini: Medico Neurologo)
- UOC Centro Parkinson e Parkinsonismi - Istituto Ortopedico Gaetano Pini-ASST Pini-CTO (Milano) (Dr.ssa Anna Zecchinelli: Medico Neurologo; Dr.ssa Nicoletta Meucci: Medico Neurologo; Dr. Claudio Mariani: Medico Neurologo; Dr. Giorgio Sacilotto: Medico Neurologo; Dr.ssa Paola Soliveri: Medico Neurologo; Dr.ssa Michela Zini: Medico Neurologo; Dr.ssa Francesca del Sorbo: Medico Neurologo; Dr.ssa Daniela Calandrella: Medico Neurologo Fondazione Grigioni).

Razionale

La Malattia di Parkinson (MP) e i parkinsonismi atipici sono caratterizzati dalla perdita di neuroni dopaminergici nella substantia nigra e in altre aree cerebrali e dalla presenza di neuriti di Lewy (LNs) nei processi cellulari e di corpi di Lewy (LBs) nel soma dei neuroni. Alcuni tra i più diffusi parkinsonismi atipici come la Paralisi Sopranucleare Progressiva e la Sindrome Corticobasale si caratterizzano per la presenza

all'interno delle cellule nervose di grovigli neurofibrillari composti in gran parte dalla forma iperfosforilata della proteina "Tau".
La patologia si innesca almeno 20 anni prima del manifestarsi dei primi sintomi, questa fase è detta prodromica, per questo motivo è di urgente interesse individuare biomarcatori di malattia precoci, specifici e facilmente accessibili. La presenza di un "Disturbo del comportamento nella fase REM- [RBD = REM sleep Behavior Disorder]" (caratterizzato da verbalizzazioni e movimenti, talvolta aggressivi (es: sbracciarsi, colpire, scalciare) che si verificano durante il sonno REM) può anticipare l'esordio della Malattia di Parkinson di circa 15-20 anni. Per tale motivo è di fondamentale importanza studiare questa tipologia di pazienti per l'identificazione di marcatori validi e affidabili nel monitorare la progressione della neurodegenerazione e la conversione verso una forma di MP conclamata.
La maggior componente dei LNs e dei LBs è la forma aggregata e citotossica della proteina presinaptica α-sinucleina (α-Syn). Non sono ancora noti i processi attraverso cui l'α-Syn inizia ad aggregare e causare neurotossicità. Oltre che nei LBs, l'α-Syn si accumula negli assoni e nel terminale presinaptico dei neuroni, suggerendo che questi accumuli fungano da innesco per la sinaptopatia che si osserva nella MP. A livello presinaptico, l'α-Syn è disponibile fisiologicamente nella sua conformazione nativa di monomero solubile (Burrè E, 2015) dove interagisce con il complesso proteico SNARE (costituito dalle proteine VAMP-2, SNAP-25 e Syntaxina-1). SNARE viene assemblato a livello presinaptico migliaia di volte al minuto permettendo il processo di esoscitosi delle vescicole sinaptiche contenenti i neurotrasmettitori. Sebbene non sia ancora stata del tutto chiarita la funzione fisiologica di α-Syn, è però noto che funga da *chaperone* nel promuovere l'assemblaggio di SNARE attraverso un legame diretto con VAMP-2 e i fosfolipidi presenti sulle vescicole sinaptiche (Bridi JC et al., 2018).
Con il termine vescicole extracellulari (EVs) si intendono esosomi e microvescicole, ovvero nanoparticelle delimitate da un doppio strato fosfolipidico che si differenziano sia per la dimensione, sia per la modalità con cui originano dalla cellula. Le EVs partecipano alla comunicazione intercellulare trasportando molecole bioattive quali proteine e acidi nucleici dalla cellula di origine alle cellule recipienti dove esplicano la loro funzione biologica; possono essere isolate da quasi tutti i fluidi biologici quali il sangue, le urine, la saliva, il liquido seminale, il liquido amniotico, il liquido sinoviale, il liquido cefalorachidiano e il latte materno (Urbanelli L et al., 2013). Per le loro piccole dimensioni, dell'ordine dei nanometri, sono in grado di attraversare la barriera ematoencefalica (BBB dall'inglese 'blood-brain barrier') in entrambe le direzioni (Wiklander OP et al., 2015; Bala S et al., 2013; Chen CC et al., 2016). Una recente metodica utilizzata anche dal nostro gruppo (Goetzl EJ et al., 2016) permette di isolare dal sangue periferico EVs di origine neurale (NDEs) utilizzando un anticorpo diretto verso L1CAM (CD171), proteina di membrana espressa dai neuroni a livello cerebrale. Le NDEs così isolate possono quindi essere studiate nel loro contenuto allo scopo di

individuare biomarcatori periferici facilmente accessibili, indicatori di patologie del sistema nervoso centrale (SNC).

Il nostro gruppo ha recentemente dimostrato che la proteina SNAP-25 è presente nelle NDEs. Il dosaggio di SNAP-25 nelle NDEs è stato effettuato su pazienti affetti da Malattia di Alzheimer (MA) e su soggetti sani di controllo (HC) ed è stato dimostrato che nei pazienti AD le concentrazioni di SNAP-25 sono inferiori rispetto ai controlli sani (Agliardi C et al., 2019). Questi risultati sono in linea con quelli derivanti dalle misurazioni autoptiche di SNAP-25 nel cervello dove si riscontrano infatti ridotte concentrazioni di SNAP-25 nei cervelli dei pazienti AD rispetto ai soggetti sani. Ciò indicherebbe che la misurazione periferica di SNAP-25 potrebbe essere un indice di integrità sinaptica di facile accesso che rispecchia quello che avviene a livello cerebrale durante la patologia.

Il 99% dell'α-Syn che si rileva nel sangue deriva dagli eritrociti e dalle piastrine (Shi M et al., 2010), per questo motivo la valutazione della sola α-Syn di origine neurale attraverso lo studio delle EVs periferiche risulta essere così interessante. Ad oggi, pochi studi hanno valutato l'α-Syn nelle NDEs ed i risultati sono contraddittori. Shi et al. ha correlato l'α-Syn monomerica con la severità di malattia (Shi M et al., 2014). Zhao et al. riporta dosaggi di α-Syn monomerica più alti nei pazienti con MP rispetto ai controlli (Zhao ZH et al., 2018). Un unico recente lavoro ha valutato l'α-Syn oligomerica nelle NDEs e ha evidenziato al contrario valori più bassi nei pazienti con MP rispetto ai controlli. Va evidenziato che i pazienti con MP di questo lavoro erano tutti in una fase precoce di malattia (Si X et al, 2019).

Obiettivi

- **Obiettivo principale:** individuazione di biomarcatori periferici diagnostici e prognostici nella MP, nei soggetti con Disturbo del comportamento nella fase REM e nei parkinsonismi atipici, dosabili nellevescicole esosomiali di natura neurale (dopaminergica e serotoninergica) e gliale.
- **Obiettivo secondario:** valutazione del fattore predittivo di tali biomarcatori nella risposta al trattamento riabilitativo e nella valutazione della sua efficacia nella MP.

Traslazionalità

Per la MP, per il Disturbo del comportamento nella fase REM e per gli altri parkinsonismi atipici c'è un urgente bisogno di individuare biomarcatori di malattia specifici, precoci, validi e facilmente accessibili. I sintomi clinici sono spesso non specifici e tardivi e i trattamenti di intervento clinico e farmacologico e riabilitativo

dovrebbero idealmente essere iniziati appena possibile quando i sintomi si evidenziano o, meglio ancora, quando i biomarcatori possono confermare una malattia ancora apparentemente silente al fine di bloccare o ridurre danni irreversibili alle sinapsi e ai neuroni.

L'α-Syn oligomerica, la proteina tau in stato fosforilato e le proteine del complesso SNARE negli esosomi neurali periferici potrebbero essere candidati biomarcatori precoci di malattia.

Inoltre tali biomarcatori potrebbero rappresentare degli indicatori "periferici" attendibili del grado di accumulo della α-Syn neuro-tossica e in ultima analisi potrebbero essere utilizzati come "parametri di efficacia" nei trattamenti riabilitativi per la MP nei diversi stadi di severità.

I principali outcome riabilitativi che verranno messi in correlazione con i biomarcatori saranno le modificazioni dei punteggi delle principali scale motorie (es: UPDRS) non motorie (es: non motor symptoms scale…etc) e neuro-psico-comportamentali (es. MoCA Test, Mini-Mental Parkinson, etc).

Metodologia

Disegno dello studio

Lo studio si dividerà in due parti: la prima parte dello studio è osservazionale/trasversale (*cross-sectional*) multicentrica e consisterà nell'ottenere campioni di sangue di soggetti con MP, parkinsonismi atipici e RBD; la seconda parte dello studio, interamente svolta presso il Centro S. Maria Nascente della Fondazione Don C. Gnocchi, è sperimentale (trial clinico) e consisterà nel sottoporre i soggetti con MP ad un trattamento riabilitativo (vedi *"procedure nel dettaglio"*).

Tipo di studio osservazionale

Studio multicentrico, cross-sectional.

I soggetti partecipanti verranno arruolati presso i seguenti Centri:

1) Ambulatorio Parkinson di II° livello; Macroattività Ambulatoriale Complessa; Reparto diNeurologia Riabilitativa del Centro IRCCS S. Maria Nascente della Fondazione Don C. Gnocchi di Milano.
2) Unità di Neurologia, Neurofisiologia, Neurobiologia; Dipartimento di Medicina; Università Campus Bio-Medico di Roma (Dott. Massimo Marano; Medico Neurologo)
3) Fondazione IRCCS Ca' Granda Ospedale Maggiore Policlinico - Dipartimento delle Neuroscienze e Salute Mentale- Unità di Neurologia - (Dott. Alessio Di Fonzo; Medico Neurologo)
4) Azienda Ospedaliero-Universitaria di Cagliari - Unità Operativa Complessa Neurologia- Centro del sonno (Prof.ssa Monica Puligheddu: Medico Neurologo; Dr. Mario Meloni: Medico Neurologo, PhD).
5) Azienda Ospedaliero-Universitaria di Padova; Dipartimento di neuroscienze-Clinica Neurologica (Prof.ssa Miryam Carecchio; Medico Neurologo; Prof. Angelo Antonini; Medico Neurologo)

6) UOC Centro Parkinson e Parkinsonismi - Istituto Ortopedico Gaetano Pini-ASST Pini-CTO (Milano) (Dr.ssa Anna Zecchinelli; Medico Neurologo; Nicoletta Meucci, Medico Neurologo; Claudio Mariani, Medico Neurologo; Giorgio Sacilotto, Medico Neurologo; Paola Soliveri, Medico Neurologa; Michela Zini, Medico Neurologa; Francesca del Sorbo, Medico Neurologa; Daniela Calandrella, Medico Neurologa Fondazione Grigioni.

Ciascun soggetto partecipante che avrà sottoscritto il Consenso Informato sarà sottoposto a prelievo ematico presso il proprio Centro di arruolamento; quindi i campioni ematici saranno processati presso il Laboratorio di Medicina molecolare e Biotecnologie dell'IRCCS S. Maria Nascente della Fondazione Don Gnocchi.

Tipo di studio sperimentale

Studio monocentrico, controllato e randomizzato.

I soggetti partecipanti verranno arruolati presso il seguente Centro:

Ambulatorio Parkinson di II° livello, Macroattività Ambulatoriale Complessa e Reparto di Neurologia Riabilitativa del centro IRCCS S. Maria Nascente della Fondazione Don C. Gnocchi.

Gli esperimenti verranno effettuati sui campioni di sangue dei soggetti partecipanti e processati presso il Laboratorio di Medicina molecolare e Biotecnologie della Fondazione Don Gnocchi.
I pazienti arruolati verranno sottoposti a trattamenti riabilitativi (*vedi "Procedure*").

Casi/controlli

Per partecipare allo studio tutti i soggetti arruolati dovranno aver firmato un consenso informato approvato dal Comitato Etico della Fondazione Don C. Gnocchi e dai Comitati Etici dei rispettivi Centri partecipanti.
Ci si prefigge di arruolare:

- 60 pazienti affetti da MP (alfa-sinucleinopatia);
- 80 pazienti affetti da Paralisi Sopranucleare Progressiva (PSP)/Sindrome Corticobasale (SCB)(Taupatia);
- 60 pazienti affetti da Atrofia Multisistemica (AMS) (alfa-sinucleinopatia);
- 30 pazienti affetti da Disturbo del Comportamento nella fase REM (alfa-sinucleinopatia).
- 40 soggetti sani di controllo (HC), paragonabili per età e sesso ai pazienti reclutati.

I campioni ematici dei pazienti affetti da PSP/SCB/AMS dovranno pervenire dai seguenti Centri:

- Unità di Neurologia, Neurofisiologia, Neurobiologia; Dipartimento di Medicina; Università Campus Bio-Medico di Roma (Dott. Massimo Marano; Medico Neurologo)
- Fondazione IRCCS Ca' Granda Ospedale Maggiore Policlinico - Dipartimento delle Neuroscienze e Salute Mentale- Unità di Neurologia - (Dott. Alessio Di Fonzo; Medico Neurologo)

- Azienda Ospedaliero-Universitaria di Padova; Dipartimento di neuroscienze-Clinica Neurologica (Prof.ssa Miryam Carecchio; Medico Neurologo; Prof. Angelo Antonini; Medico Neurologo).
- UOC Centro Parkinson e Parkinsonismi - Istituto Ortopedico Gaetano Pini-ASST Pini-CTO (Milano) (Dr.ssa Anna Zecchinelli; Medico Neurologo; Nicoletta Meucci, Medico Neurologo; Claudio Mariani, Medico Neurologo; Giorgio Sacilotto, Medico Neurologo; Paola Soliveri, Medico Neurologa; Michela Zini, Medico Neurologa; Francesca del Sorbo, Medico Neurologa; Daniela Calandrella, Medico Neurologa Fondazione Grigioni.

Ogni Centro dovrà far pervenire un totale di 20 campioni di sangue di pazienti con PSP/SCB e 15 campioni di sangue di pazienti con AMS.

I campioni di sangue dei pazienti affetti da RBD dovranno pervenire dal seguente Centro:

- Azienda Ospedaliero- Universitaria di Cagliari - Unità Operativa Complessa Neurologia- Centro del sonno (Prof.ssa Monica Puligheddu: Medico Neurologo; Dr. Mario Meloni: Medico Neurologo, PhD).

Procedure

Da ciascun soggetto partecipante allo studio verrà prelevato un campione di sangue periferico (37 ml) che verrà successivamente separato ed il cui siero sarà conservato ad una temperatura di -80°C fino all'utilizzo. Dal siero verranno isolate le EVs di origine neurale mediante una metodica a doppio step che prevede prima l'utilizzo del polimero commerciale ExoQuick® per l'isolamento degli esosomi totali e in seguito l'arricchimento dei soli esosomi di origine neurale mediante anticorpo biotinilato contro la molecola neurale L1CAM (CD171) ed una resina con streptavidina (Mustapic M et al., 2017). Verranno quindi estratte le proteine dalle EVs così isolate che verranno utilizzate in saggi ELISA per il dosaggio delle proteine: α-Syn oligomerica, SNAP-25, syntaxina-1, VAMP-2, Tau fosforilata). Verranno inoltre effettuati esperimenti di Western-blotting per valutare le interazioni tra α-Syn oligomerica e le proteine del complesso SNARE. Si cercherà inoltre di mettere a punto una nuova metodica per poter isolare extravescicole provenienti dai neuroni dopaminergici/serotoninergici e dalle cellule gliali medianti anticorpi specifici delle cellule d'origine.

La seconda parte dello studio (studio interventistico) verrà condotta esclusivamente presso l'IRCCS S. Maria Nascente della Fondazione Don C. Gnocchi. Tutti i soggetti arruolati presso l'Ambulatorio Parkinson della Fondazione Don Gnocchi che rispetteranno i criteri di inclusione, dopo aver letto il Foglio Informativo e sottoscritto il Consenso Informato, riceveranno la valutazione iniziale e saranno sottoposti al prelievo di sangue (T0) e saranno quindi randomizzati tramite una lista di randomizzazione pre-compilata in un gruppo sperimentale ed in un gruppo di controllo.

Il gruppo sperimentale verrà incluso in un programma riabilitativo intensivo e multidisciplinare per il recupero e il potenziamento della funzionalità motoria e "non-motoria" con particolare riferimento alla riabilitazione cognitiva.
Il gruppo di controllo verrà incluso in un programma di auto-trattamento domiciliare.

Procedure nel dettaglio

Trattamento riabilitativo sperimentale

Il setting riabilitativo del gruppo sperimentale sarà quello convenzionale in clinica (Macroattività Ambulatoriale Complessa-MAC). Il programma riabilitativo avrà durata di 6 settimane consecutive. Il trattamento riabilitativo prevede l'esecuzione di 30 sedute/5 giorni a settimana della durata di 160'/die ciascuna (80' riabilitazione motoria; 40' riabilitazione cognitiva e 40' riabilitazione logopedica) per 3 giorni/settimana e 180'/die (80' riabilitazione motoria; 60' riabilitazione cognitiva e 40' riabilitazione logopedica) per 2 giorni/settimana. All'interno dell'intervento di riabilitazione motoria saranno proposte 18 sedute (3 volte alla settimana) in cui è previsto un lavoro aerobico su treadmill della durata di 20 minuti, esercizi di equilibrio e rinforzo funzionale (20 minuti).
Le rimanenti sedute di riabilitazione motoria saranno di tipo convenzionale comprendenti esercizi definiti dal terapista secondo le necessità terapeutiche del paziente.
Per quanto concerne il trattamento di riabilitazione o potenziamento cognitivo, i soggetti del gruppo sperimentale saranno sottoposti ad un programma personalizzato sviluppato e supervisionato da un neuropsicologo; tale trattamento sarà rivolto principalmente alle funzioni frontali/esecutive/attentive/visuo-spaziali-costruttive oltre che alle funzioni mnesiche e linguistiche e sarà proposto sia in modalità tradizionale (3 volte/settimana) sia attraverso il coinvolgimento in attività condotte con il supporto della realtà virtuale semi-immersiva "Virtual Reality Rehabilitation System" (VRRS) (2 volte/settimana) tramite l'utilizzo di stimoli interattivi per offrire l'opportunità di percepire ed interagire, nel modo più reale possibile, con uno stimolo o un oggetto virtuale, ed avere feedback immediati secondo diversi canali (visivo, uditivo, propriocettivo, vestibolare ed in alcuni casi anche tattile). Il trattamento VRRS è strutturato in 2 sedute alla settimana della durata di 60 minuti ciascuna per 6 settimane consecutive, per un totale di 12 sedute. Nello specifico, il trattamento VRRS comprende esercizi progettati per migliorare le funzioni esecutive, le capacità visuo-spaziali, l'attenzione e la memoria (per approfondimenti sulla strumentazione si veda il sito dell'azienda produttrice al seguente link: http://khymeia.com/prodotti/vrrs/).
I soggetti del gruppo sperimentale saranno sottoposti ad un programma personalizzato e supervisionato da un logopedista che comprenderà la valutazione ed il trattamento della voce (disfonia), dell'articolazione (disartria) e della deglutizione (disfagia). La valutazione sarà composta da una valutazione strumentale, dall'autovalutazione e dalla valutazione clinica. Verranno utilizzate tecniche innovative sia per la valutazione

(analisi acustica dello speech) sia per il trattamento (biofeedback con Vitalstim). Verranno inoltre fornite indicazioni alimentari per una corretta deglutizione e verranno proposti al paziente e al caregiver momenti di counselling (ad esempio per l'addestramento all'utilizzo di prodotti ad hoc per la disfagia). Inoltre i soggetti che non presentano deficit verranno coinvolti in una breve formazione per prevenire i disturbi di voce e deglutizione.

Trattamento riabilitativo di controllo

I soggetti del gruppo di controllo saranno sottoposti ad un programma di auto-trattamento per 40'/die che consiste in esercizi di *stretching* e di mobilizzazione attiva a domicilio. Prima di iniziare il programma i soggetti verranno sottoposti ad una seduta educazionale e di training con il fisioterapista per apprendere la corretta modalità di svolgimento degli esercizi proposti. Ai soggetti verranno consegnate le istruzioni dettagliate degli esercizi e un diario dove dovranno essere annotati gli esercizi effettuati, gli eventuali effetti collaterali e/o le difficoltà specifiche incontrate durante il programma. L'aderenza al trattamento verrà monitorata una volta a settimana tramite contatto telefonico.

I soggetti del gruppo sperimentale e del gruppo di controllo saranno sottoposti alle valutazioni e al prelievo di sangue prima di iniziare il trattamento (T0), alla fine del trattamento (T1) e dopo 3 mesi dalla fine del trattamento (T2).

Indagini molecolari

- Misurazione delle concentrazioni di α-Syn oligomerica e delle proteine del complesso SNARE (SNAP-25, sintaxina-1, VAMP-2) nelle vescicole extracellulari periferiche di origine neurale (NDEs) isolate dai pazienti in diversi stadi di malattia e di controlli sani;
- Valutazione delle interazioni molecolari tra α-Syn oligomerica e le proteine del complesso SNARE nelle NDEs;
- Messa a punto di una metodica per poter isolare nel siero, mediante anticorpi specifici, vescicole extracellulari originatesi dai soli neuroni dopaminergici e dalle cellule della glia/microglia;
- Messa a punto di una metodica per poter isolare nel siero, mediante anticorpi specifici, vescicole extracellulari originatesi dai soli neuroni dopaminergici e dai soli neuroni serotoninergici e noradrenergici.

- Misurazione delle concentrazioni di α-Syn oligomerica e delle proteine del complesso SNARE nelle NDEs prima e dopo trattamenti riabilitativi;
- Studio della correlazione tra i livelli di α-Syn oligomerica nelle NDEs e le due principali forme cliniche di MP: forma rigido-acinetica versus forma tremorigena;
- Misurazione della concentrazione di α-Syn oligomerica e della Tau fosforilata negli esosomi neurali/gliali e studio del relativo rapporto nella MP, nei parkinsonismi atipici (atrofia multisistemica; paralisi sopranucleare progressiva e sindrome cortico-basale) e RBD e valutazione del potenziale ruolo nella diagnostica differenziale;
- Studio del rapporto tra α-Syn oligomerica di origine neurale (neuroni dopaminergici), α-Syn oligomerica di origine microgliale e eventuali modificazioni nei vari stadi di malattia.

Valutazioni cliniche, motorie e funzionali

Misurazione dei principali parametri clinici (sintomatologia motoria e non motoria) ai tempi T0, T1 e T2 tramite scale e questionari specifici per la MP e parkinsonismi atipici:

1) Valutazioni cliniche neurologiche e neuro-psico-patologiche:

A) Valutazioni neurologiche:

- Informazioni anamnestiche (modalità e data di esordio; data diagnosi; presenza/descrizione esami strumentali; presenza attuale di segni e sintomi);
- Terapia farmacologica in atto ed eventuali modifiche (date modifiche tp, dosaggi -per calcolo ledd- e orari assunzione tp).
- Somministrazione e raccolta informazioni per compilazione delle seguenti scale:
    - Modified Hoehn and Yahr Scale (Modified H&Y) (Goetz et al. 2004)
    - Movement Disorders Society Unified Parkinson's disease rating scale Part I-IV (MDS-UPDRS Part I-IV) (Antonini et al. 2013)
    - Non-Motor Symptoms Scale (NMSS) (Chaudhuri et al., 2007; Cova et al., 2017);
    - Composite Autonomic Symptoms Score COMPASS 31 (Pierangeli et al. 2015)
    - Modified Barthel Index (MBI) (Galeoto et al. 2015)
    - Modified Cumulative Illness Rating Scale (CIRS) (Salvi et al., 2008)
    - Numeric Rating Scale (NRS) (Hartrick et al., 2003)
    - 39-item Parkinson's Disease Questionnaire (PDQ-39) (Galeoto et al., 2018);
    - Parkinson Fatigue Scale (PFS) (Brown et al., 2005; Siciliano et al., 2019);
    - Barratt Impulsiveness Scale-11 (BIS-11) (Fossati et al., 2001);
    - Questionnaire for Impulsive-Compulsive Disorders in Parkinson's disease (QUIP-RS-IT) (Weintraub et al., 2009).
    - Pittsburgh Sleep Quality Index (PSQI) (Curcio et al. 2013)
    - Parkinson Sleep Scale (PSS) (Pellecchia et al 2012);
    - Epworth Sleepiness Scale (ESS) (Vignatelli et al. 2003);
    - REM sleep behavior disorder screening questionnaire (RBDSQ) (Marelli et al., 2016).

B) Valutazioni neuropsicologiche e psico-comportamentali:

1) I Livello - scale cognitive:
    - Montreal Cognitive Assessment (MoCA Test) (Conti et al., 2014; Santangelo et al., 2014);
    - Test del Breve Racconto (vers. Babcock '6 Dicembre'; Carlesimo et al., 2002)

- Trail Making Test (TMT) (Giovagnoli et al., 1996)
- Matrici di Raven CPM-47 (Caltagirone et al, Carlesimo et al, 1996)
- Span verbale Forward e Backward (Monaco et al., 2013)
- Copia e Rievocazione Figura Di Rey (Caffarra et al., 2002)
- Fluenza Alternata (Costa et al., 2014)
- Frontal Assessment Battery (FAB) (Appollonio et al., 2005)
- Stroop Test-Versione Breve (Caffarra et al., 2002)
- Mini-Mental Parkinson (MMP) (Costa et al., 2013)
- Boston Naming Test (shortened version) (Mack et al., 1992)

2) I Livello – Scale neuropsichiatriche/funzionali/Qualità di Vita (QoL) (alcune autocompilate dal paziente, altre somministrate e compilate dal caregiver):

- Cognitive Reserve Index-questionnaire (CRI-q) (Nucci et al., 2011)
- Beck Depression Inventory-II (BDI-II) (Beck et al., 1996; Ghisi et al., 2006) (autocompilato dal paziente)
- State-Trait Anxiety Inventory. Forma Y (STAI-Y) (Spielberger et al., 1983; Pedrabissi & Santinello, 1989) (autocompilato dal paziente)
- Indice di autonomia nelle attività della vita quotidiana (Activities of Daily Living, ADL) (Katz et al., 1963) (compilato dal caregiver)
- Indice di autonomia nelle attività strumentali della vita quotidiana (Instrumental Activities Of Daily Living, IADL) (Lawton et al., 1969) (compilato dal caregiver)
- Functional Activities Questionnaire (FAQ) (Pfeffer et al., 1982; versione Sansotera e Foderaro, 2007) (compilato dal caregiver)
- Caregiver Burden Inventory (CBI) (Novak e Guest, 1989) (compilato dal caregiver)
- NeuroPsychiatric Inventory Questionnaire (NPI-Q) (Kaufer et al., 2000) (compilato dal caregiver)

3) II Livello - scale cognitive:

- Clock Drawing Test (CDT) (Mondini et al., 2003)
- Free and Cued Selective Reminding Test (FCSRT) (Frasson et al., 2011)
- Span visuo-spaziale (Test di Corsi) Forward e Backward (Monaco et al., 2013)
- Matrici attentive (Spinnler e Tognoni, 1987)
- Symbol Digit Modalities Test (SDMT) (Vers. Orale) (Nocentini et al., 2006)
- Test Di Imitazione Di Gesti (versione: De Renzi et al., 1968; taratura: De Renzi et al., 1980, 1986)
- Weigl's Sorting Test (versione e taratura: Spinnler e Tognoni, 1987)

4) II Livello - Scale neuropsichiatriche/funzionali/Qualità di Vita (QoL) (alcune autocompilate dal paziente, altre somministrate e compilate dal caregiver):

- Dimensional Apathy Scale (I-DAS) (Santangelo et al., 2017) (autocompilato dal paziente)
- Snaith-Hamilton Pleasure Scale (SHAPS) (Santangelo et al., 2009) (autocompilato dal paziente)

- Toronto Alexithymia Scale (TAS-20) (Taylor et al., 1992; Bressi et al., 1996)

C) Valutazioni motorie e funzionali (le valutazioni motorie verranno somministrate circa un'ora dopo la prima presa giornaliera del farmaco dopaminergico (levodopa; dopamino-agonista etc)):

- Timed-Up and -Go Test (TUG) per valutare l'equilibrio dinamico
- Modified Dynamic Gait Index (mDGI) per valutare l'equilibrio dinamico;
- 6 Minute Walk test (6MWT) per rilevare la resistenza nel cammino;
- 10 meter walk test per valutare la velocità del cammino;
- 5-time sit-to-stand per valutare la forza degli arti inferiori;
- Raccolta retrospettiva del numero di cadute negli ultimi 2 mesi
- Analisi del cammino, tramite sistema optoelettronico, ad esaurimento nel quale i soggetti cammineranno in maniera continuativa all'interno del laboratorio fino al raggiungimento di uno stato di fatica percepito di 17 (sforzo "molto pesante") sulla scala modificata di BORG. Lo stato di affaticamento sarà monitorato ogni minuto per indagare le variazioni di parametri legati al cammino sensibili all'affaticamento.

Valutazione strumentale mediante dispositivo indossabile (wearable):

I soggetti del gruppo sperimentale e del gruppo di controllo saranno sottoposti a monitoraggio actigrafico nella settimana precedente l'inizio del trattamento (T0), nella settimana successiva alla fine del trattamento (T1) e nell'ambito della valutazione di follow-up a tre mesi (T2). I soggetti indosseranno un actigrafo a ciascun polso con l'obiettivo di raccogliere informazioni quantitative relativamente all'attività motoria. Ciascun soggetto durante i giorni in cui avrà gli actigrafi sarà tenuto alla compilazione di un diario delle abitudini e attività motorie oltre alla registrazione dell'assunzione della terapia farmacologica. L'actigrafo, dall'aspetto di un braccialetto/orologio (https://www.geneactiv.org/actigraphy/geneactiv- original/presente marchiatura CE 2004/108/EC; tested to BS EN 61000-6-1 :2007 and BS EN 61000-6-3 :2007) di seguito illustrato, permette appunto la registrazione, in modo automatico, di una serie di informazioni, che successivamente, attraverso la connessione ad un pc, possono essere scaricate, estratte ed elaborate secondo algoritmi definiti a livello ingegneristico per ricavare, ad esempio, grafici che rappresentano l'alternarsi di attività/riposo, eventuali disturbi del sonno, tremore (presente/assente, unilaterale/bilaterale), ed altre informazioni relative alla attività motoria.
Al fine di calibrare e tarare il metodo actigrafico (Rabuffetti et al 2016) a individui con MP si svolgerà una sperimentazione di laboratorio che includa prove motorie prefissate e controllate (quali cammino rettilineo a diverse velocità, azionamento di ergometro per arti superiori in assenza di carico applicato a velocità prefissata e controllata, prove funzionali standardizzate quale test TUG). È previsto che tale sperimentazione pilota coinvolga non meno di tre persone con caratteristiche compatibili al reclutamento nello studio principale.

Per monitorare l'aderenza e la partecipazione attiva al trattamento riabilitativo sia ai soggetti del gruppo di controllo che ai soggetti del gruppo sperimentale verrà somministrata la versione italiana della "Pittsburgh Rehabilitation Participation Scale (PRPS)" (Iosa et al 2021). La somministrazione verrà effettuata una volta a settimana sia ai soggetti del gruppo di controllo che ai soggetti del gruppo sperimentale.

Reclutamento campioni

Tutti i soggetti dovranno avere un'età compresa tra i 50 e gli 85 anni.

Ai soggetti arruolati verranno somministrate le scale per la stadiazione e per le valutazioni motorie e non motorie (disturbi del sonno; disautonomie; disturbi cognitivo-comportamentali).

Tutti i soggetti arruolati verranno sottoposti ad una accurata anamnesi farmacologica e, in particolare, verrà calcolata per ciascun soggetto la dose equivalente di levodopa secondo i criteri di Tomlinson del 2010 (Tomlinson et al., 2010).

I soggetti di controllo dovranno essere paragonabili per età e sesso ai soggetti affetti da MP, senza alterazioni cognitive né evidenze di patologie neurologiche acute o croniche in atto al momento dell'arruolamento, selezionati tra i coniugi dei pazienti stessi.

La diagnosi di MP verrà effettuata da medici neurologi secondo i criteri "MDS clinical diagnostic criteria for Parkinson's disease" (Postuma et al., 2015). Verranno arruolati i soggetti con MP con stadiazione Hoehn & Yahr da 1 a 3.

La diagnosi di Paralisi Sopranucleare Progressiva verrà effettuata secondo i criteri della Movement Disorders Society (Movement Disorder Society-endorsed PSP Study Group, 2017).

La diagnosi di Sindrome Corticobasale verrà effettuata secondo i criteri di Armstrong del 2013 (Armstrong MJ et al 2013).

La diagnosi di Atrofia Multisistemica verrà effettuata secondo i criteri di Gilman del 2008 (Gilman et al., 2008).

La diagnosi di Disturbo del comportamento nella fase REM verrà effettuata secondo i criteri della "International Classification of Sleep Disorders, Third Edition (ICSD-3)".
La dimensione campionaria iniziale di 80 soggetti con PSP, SCB; 60 con AMS e 30 con RBD si basa su una stima approssimativa del numero di pazienti che generalmente afferiscono nei rispettivi Centri partecipanti: 1) Unità di neurologia, neurofisiologia, neurobiologia; Dipartimento di Medicina; Università Campus Bio-Medico di Roma (Dr. Massimo Marano, Medico Neurologo). 2) Fondazione IRCCS Ca' Granda Ospedale Maggiore Policlinico - Dipartimento delle Neuroscienze e Salute Mentale- Unità di Neurologia - (Dr. Alessio Di Fonzo, Medico Neurologo). 3) Azienda Ospedaliero- Universitaria di Cagliari - Unità Operativa Complessa Neurologia- Centro del sonno (Prof.ssa Monica Puligheddu, Medico Neurologo; Dr. Mario Meloni, Medico Neurologo, PhD). 4) Azienda Ospedaliero-Universitaria di Padova; Dipartimento di neuroscienze-Clinica Neurologica (Prof.ssa Miryam Carecchio, Medico Neurologo; Prof. Angelo Antonini, Medico Neurologo). 5) Centro Parkinson e Parkinsonismi - Istituto Ortopedico Gaetano Pini-ASST Pini-CTO (Milano) – (Dr.ssa Anna Zecchinelli, Medico Neurologo).
In letteratura non esistono ad oggi studi clinici di efficacia del trattamento riabilitativo sui biomarcatori in studio (es: α-sinucleina, complesso proteico SNARE etc) pertanto la dimensione campionaria è stata calcolata sulla base di risultati preliminari di uno studio cross-sectional (Agliardi et al., 2021) condotto su 32 pazienti reclutati all'interno del Centro Parkinson di Fondazione Don Gnocchi. Pertanto considerando 2 gradi di libertà, una potenza campionaria β dell'80% e α al 5% e una percentuale di drop-out pari al 20%, sono necessari 36 pazienti per gruppo di studio, per un totale di 72 pazienti.

Analisi statistiche

I soggetti arruolati dovranno essere comparabili per età e sesso. Per valutare la normalità delle distribuzioni delle variabili continue verranno adottati i test Shapiro-Wilk e Kolmogrov-Smirnov. Per il confronto tra gruppi verranno utilizzati i test t-student e ANOVA nel caso di distribuzione normale e di Kruskal-Wallis e Mann Whitney per distribuzioni non-normali. La possibile validità del dosaggio delle proteine esosomiali sopra elencate quale biomarcatore per la MP verrà valutata mediante analisi ROC (Receiver Operating Characteristic) con il calcolo dell'area sotto la curva (AUC), la sensibilità, la specificità e l'intervallo di confidenza del 95%. In tutti i casi verrà valutato significativo un valore di $p \leq 0.05$.
Tramite le analisi di regressione logistica verranno studiate le associazioni tra le variabili indipendenti (variabili demografiche; caratteristiche fenotipiche del parkinsonismo, compresi i dati delle scale motorie e non motorie; stadiazione; LEDD; outcome riabilitativi) e la variabile dipendente (biomarcatori sierici-α-Syn oligomerica e delle proteine del complesso SNARE).

Criticità

La criticità più importante potrebbe essere la scarsa adesione al protocollo, per mancato consenso, dei pazienti. A tal proposito, verrà eseguito un controllo della numerosità alla fine del primo anno.
Non ci sono criticità legate all'utilizzo della strumentazione (actigrafo) poiché verranno utilizzati dispositivi e sistemi di misura commerciali marcati CE di uso quotidiano.

Risultati attesi

Ci si aspetta che le concentrazioni di α-Syn oligomerica, della proteina tau fosforilata e delle proteine del complesso SNARE (e i rapporti tra loro), misurate nelle vescicole extracellulari di origine cerebrale e isolate nel sangue periferico, possano essere un biomarcatore di malattia per la MP, RBD e per i parkinsonismi atipici. Inoltre, l'eventuale correlazione con i dati clinici, strumentali e con i risultati riabilitativi permetterà di valutare il possibile ruolo predittivo dei biomarcatori in esame e l'eventuale utilizzo degli stessi come "parametri di efficacia" riabilitativa nella MP.
In particolare ci si aspetta che il trattamento riabilitativo possa modulare le concentrazioni della α-Syn oligomerica e delle proteine del complesso SNARE.

Fasi del Progetto

	Tempo di Realizzazione		
**Tipologia di attività**	**Dal**	**Al**	**Responsabile**
Arruolamento pazienti e prelievo campioni biologici	Gennaio 2020	Giugno 2024	Francesca Lea Saibene; Pietro Arcuri; Anna Castagna; Elisabetta Farina; Margherita Alberoni; Elena Calabrese; Anna Salvatore
Tabulazione dati e analisi statistiche intermedie	Febbraio 2020	Settembre 2023	Franca Guerini; Cristina Agliardi
Analisi di Biologia Molecolare	Febbraio 2020	Settembre 2024	Cristina Agliardi
Scrittura Abstract per partecipazione a congressi	Giugno 2020	Dicembre 2024	Francesca Lea Saibene; Mario Meloni; Franca Guerini; Cristina Agliardi
Tabulazione dati e analisi statistiche finali	Marzo 2021	Settembre 2024	Francesca Lea Saibene; Franca Guerini; Cristina Agliardi; Marco Rabuffetti (per attività actigrafica)
Scrittura lavori scientifici per riviste internazionali	Settembre 2020	Dicembre 2024	Francesca Lea Saibene; Mario Meloni; Pietro Arcuri; Franca Guerini; Cristina Agliardi; Mario Clerici

Aspetti economici

Lo studio è stato a suo tempo approvato dalla Direzione Scientifica della Fondazione nell'ambito dell'attività di Ricerca Corrente prevista per l'anno 2020. Pertanto, tutti i costi per l'esecuzione dello studio (es: materiali per i prelievi, costi delle spedizioni dei prelievi di sangue) saranno coperti con i fondi assegnati dal Ministero della Salute alla Fondazione Don Gnocchi per suddetta attività di Ricerca Corrente.

BIBLIOGRAFIA

- A.A. of S. Medicine, International Classification of Sleep Disorders 3nd edition. Diagnostic and coding manual, 2014.
- Agliardi C, Guerini FR, Zanzottera M, Bianchi A, Nemni R, Clerici M. SNAP-25 in Serum Is Carried by Exosomes of Neuronal Origin and Is a Potential Biomarcatore of Alzheimer's Disease. Mol Neurobiol. 2019Aug;56(8):5792-5798. doi:10.1007/s12035-019-1501-x.
- Agliardi C, Meloni M, Guerini FR, Zanzottera M, Bolognesi E, Baglio F, Clerici M. Oligomeric alpha-Synand SNARE complex proteins in peripheral extracellular vesicles of neural origin are biomarkers

  for Parkinson's disease. Neurobiol Dis. 2021 Jan;148:105185. doi: 10.1016/j.nbd.2020.105185. Epub 2020Nov 18.
- Armstrong MJ, Litvan I, Lang AE, et al. Criteria for the diagnosis of corticobasal degeneration. Neurology. 2013;80(5):496-503.
- Bala S, Csak T, Momen-Heravi F, Lippai D, Kodys K, Catalano D, Satishchandran A, Ambros V et al (2015)Biodistribution and function of extracellular miRNA-155 in mice. Sci Rep5:10721. https://doi.org/10.1038/srep10721
- Bridi JC, Hirth F. Mechanisms of α-Synuclein Induced Synaptopathy in Parkinson's Disease. Front Neurosci. 2018 Feb 19;12:80. doi:10.3389/fnins.2018.00080.
- Burré J. The Synaptic Function of α-Synuclein. J Parkinsons Dis. 2015;5(4):699-713. doi: 10.3233/JPD- 150642.
- Chen CC, Liu L, Ma F, Wong CW, Guo XE, Chacko JV, Farhoodi HP, Zhang SX et al (2016) Elucidation of exosome migration across the blood-brain barrier model in vitro. Cell Mol Bioeng 9(4):509– 529. https://doi.org/10.1007/s12195-016-0458-3.
- Gilman S, Wenning GK, Low PA, Brooks DJ, Mathias CJ, Trojanowski JQ, Wood NW, Colosimo C, Durr A, Fowler CJ, Kaufmann H, Klockgether T, Lees A, Poewe W, Quinn N, Revesz T, Robertson D, Sandroni
- P, Seppi K, Vidailhet M. Second consensus statement on the diagnosis of multiple system atrophy.Neurology. 2008;71:670–676.
- Goetzl EJ, Kapogiannis D, Schwartz JB, Lobach IV, Goetzl L, Abner EL, Jicha GA, Karydas AM, Boxer A,Miller BL. Decreased synaptic proteins in neuronal exosomes of frontotemporal dementia and Alzheimer's disease. FASEB J. 2016 Dec;30(12):4141-4148.
- Höglinger GU, Respondek G, Stamelou M, Kurz C, et al. Movement Disorder Society-endorsed PSPStudy Group. Clinical diagnosis of progressive supranuclear palsy: The movement disorder society criteria. Mov Disord. 2017 Jun;32(6):853-864.
- Iosa M, Galeoto G, De Bartolo D, Russo V, Ruotolo I, Spitoni GF, Ciancarelli I, Tramontano M, AntonucciG, Paolucci S, Morone G. Brain Sci. 2021 May 13;11(5):626. doi: 10.3390/brainsci11050626
- Mustapic M, Eitan E, Werner JK Jr, Berkowitz ST, Lazaropoulos MP, Tran J, Goetzl EJ, Kapogiannis D. Plasma Extracellular Vesicles Enriched for Neuronal Origin: A Potential Window into Brain Pathologic Processes. Front Neurosci. 2017 May 22;11:278. doi: 10.3389/fnins.2017.00278.
- Postuma RB, Berg D, Stern M, MDS clinical diagnostic criteria for Parkinson's disease. Mov Disord. 2015 Oct;30(12):1591-601. doi: 10.1002/mds.26424.
- Rabuffetti M, Meriggi P, Pagliari C, Bartolomeo P, Ferrarin M. Differential actigraphy for monitoring asymmetry in upper limb motor activities. Physiol Meas. 2016 Oct;37(10):1798-1812. doi: 10.1088/0967- 3334/37/10/1798.
- Shi M, Zabetian CP, Hancock AM, Ginghina C, Hong Z, Yearout D, Chung KA, Quinn JF, Peskind ER, Galasko D, Jankovic J, Leverenz JB, Zhang J. Significance and confounders of peripheral DJ-1 and alpha- synuclein in Parkinson's disease. Neurosci Lett. 2010 Aug 9;480(1):78-82. doi: 10.1016/j.neulet.2010.06.009.

- Si X, Tian J, Chen Y, Yan Y, Pu J, Zhang B. Central Nervous System-Derived Exosomal Alpha-Synuclein in Serum May Be a Biomarcatore in Parkinson's Disease. Neuroscience. 2019 Aug 10;413:308-316. doi: 10.1016/j.neuroscience.2019.05.015.
- Tomlinson CL, Stowe R, Patel S, Rick C, Gray R, Clarke CE. Systematic review of levodopa dose equivalency reporting in Parkinson's disease. Mov Disord. 2010 Nov 15;25(15):2649-53.
- Urbanelli L, Magini A, Buratta S, Brozzi A, Sagini K, Polchi A, Tancini B, Emiliani C (2013) Signaling pathways in exosomes biogenesis, secretion and fate. Genes (Basel) 4(2):152– 170. https://doi.org/10.3390/genes4020152
- Wiklander OP, Nordin JZ, O'Loughlin A, Gustafsson Y, Corso G, Mäger I et al (2015) Extracellular vesiclein vivo biodistribution is determined by cell source, route of administration and targeting. J Extracell Vesicles4:26316. https://doi.org/10.3402/jev.v4.26316.
- Zhao ZH, Chen ZT, Zhou RL, Zhang X, Ye QY, Wang YZ. Increased DJ-1 and α-Synuclein in Plasma Neural-Derived Exosomes as Potential Markers for Parkinson's Disease. Front Aging Neurosci. 2019 Jan 14;10:438. doi:10.3389/fnagi.2018.00438.

Possibili benefici per il paziente

I benefici attesi sono orientati all'acquisizione di maggiori conoscenze scientifiche sulla malattia. La partecipazione allo studio non incide sulla abituale attività assistenziale del paziente in quanto non comporta l'esclusione o la riduzione del trattamento ordinario a cui il soggetto è sottoposto.

La miglior descrizione degli aspetti motori, non-motori e cognitivi nella MP e nei Parkinsonismi potrebbe fornire informazioni utili in termini diagnostici e prognostici più dettagliate e più precise per l'ottimizzazione delle risorse in ottica riabilitativa e di continuità di cura (anche attraverso nuovi strumenti e procedure -ad es. monitoraggio dell'attività motoria con actigrafia anche domiciliare), con possibile ricaduta sul SSN per protocolli di intervento non farmacologici sulla popolazione in oggetto.

Possibili rischi / effetti collaterali per il paziente.

Il paziente non sarà esposto a nessun rischio clinicamente rilevante.

L'unica procedura potenzialmente critica per il paziente può essere il prelievo ematico, che potrebbe provocare in alcuni casi ematoma in sede di prelievo: per questo motivo durante questa fase, particolare attenzione verrà posta ai soggetti sottoposti a terapia con antiaggreganti e anticoagulanti.

Non sono individuabili possibili rischi né effetti collaterali poiché la registrazione di dati attraverso gli actigrafi (telemonitoraggio di informazioni motorie) non presenta rischi né possibili effetti collaterali.

Raccolta e trattamento dei dati.

Verranno raccolte notizie cliniche ed anamnestiche per ogni paziente come la durata malattia, il decorso della patologia, i trattamenti pregressi e le terapie in atto. Per i soggetti con la MP verranno inoltre somministrate le scale per la valutazione della sintomatologia motoria e non - motoria.

Verranno raccolti dati di carattere generale (grado di parentela, sesso e età) relativi ai Caregivers dei pazienti arruolati. Le informazioni raccolte relative ai soggetti reclutati e ai caregivers avranno un ruolo fondamentale nelle analisi statistiche, verranno pertanto utilizzate come covariate durante tali analisi. Tutti i dati anamnestici,

clinici (valutazioni prima e dopo i trattamenti riabilitativi) e biologici verranno raccolti in un database anonimizzato tramite codice numerico progressivo e valutati come dati aggregati accessibili unicamente dai ricercatori partecipanti.
