# Supplementary material for: Investigating the effects of a daily multidisciplinary intensive outpatient rehabilitation program on innovative biomarkers in people with Parkinson’s disease: Study protocol for a phase III randomized controlled clinical trial
Source: PLoS One. 2024 Oct 23;19(10):e0309405. doi: 10.1371/journal.pone.0309405 (PMC11498734; doi:10.1371/journal.pone.0309405)
Supplement: S2 Protocol — Clinical trial protocol_English Version (Ethics approved). (PDF) [file pone.0309405.s005.pdf]

STUDY PROTOCOL

Study title:

"Characterization of extracellular vesicles of neural and glial origin for the identification of reliable molecular biomarkers for Parkinson's Disease (PD), atypical Parkinsonisms, and Idiopathic REM sleep Behavior Disorder."

Study Principal Investigator

Dr. Francesca Lea SAIBENE

Study design

Interventional, cross-sectional, experimental, randomized controlled trial.

Interventions

Blood sample collection. Blood samples will be processed at the Laboratory of Molecular Medicine and Biotechnologies to obtain serum from which extracellular vesicles (EVs) will be isolated for biological investigations outlined in the study.

Rehabilitative treatments, administration of scientifically validated tests for assessing motor and non-motor functions, motor monitoring through actigraphs.

Expected duration

From April 1, 2020, to December 31, 2024

Participating unities and responsible researchers

Research group:

- Laboratory of Molecular Medicine and Biotechnologies, IRCCS Don Gnocchi Foundation Santa Maria Nascente (Dr. Cristina Agliardi: Biotechnologist researcher; Dr. Franca Rosa Guerini: Biologist researcher; Prof. Mario Clerici: Scientific Director);
- Rehabilitation Neurology Unit; I° and II° level Parkinson Outpatient Clinic (MAC Parkinson), IRCCS Don Gnocchi Foundation Santa Maria Nascente (Dr. Francesca Lea Saibene: Psychologist, Neuropsychologist, Psychotherapist, Researcher; Dr. Anna Salvatore: Psychologist, Researcher; Dr. Pietro Arcuri: Physiatrist; Dr. Anna Castagna: Neurologist; Dr. Elisabetta Farina: Neurologist, PhD; Dr. Margherita Alberoni: Neurologist; Dr. Elena Calabrese: Neurologist);

- Walking and Balance Research Laboratory (LaRiCE) IRCCS Don Gnocchi Foundation Santa Maria Nascente (Dr. Davide Cattaneo: FKT, PhD; Dr. Thomas Bowman: FKT, PhD; Dr. Denise Anastasi: FKT);
- Technological Pole - IRCCS Don Gnocchi Foundation Santa Maria Nascente (Eng. Maurizio Ferrarin: Biomedical Eingeneer; Eng. Marco Rabuffetti: Biomedical Eingeneer; Eng. Tiziana Lencioni: Biomedical Eingeneer; Eng. Ilaria Carpinella: Biomedical Eingeneer);
- Advanced Diagnostic and Rehabilitation Therapy Center (CADiTeR) IRCCS Don Gnocchi Foundation Santa Maria Nascente (Dr. Francesca Baglio: Neurologist; Dr. Chiara Pagliari: Psychologist, Researcher);
- Neurology, Neurophysiology, Neurobiology Unit; Department of Medicine; Campus Biomedico University of Rome (Dr. Massimo Marano: Neurologist);
- Fondazione IRCCS Ca' Granda Ospedale Maggiore Policlinico – Department of Neurosciences and Mental Health – Neurology unit – Sleep Center (Dr. Alessio Di Fonzo: Neurologist);
- University Hospital of Cagliari – Complex Unit of Neurology – Sleep Center (Prof. Monica Puligheddu: Neurologist; Dr. Mario Meloni: Neurologist, PhD);
- University Hospital of Padova; Department of Neuroscience – Neurological Clinic (Prof. Miryam Carecchio: Neurologist; Prof. Angelo Antonini: Neurologist);
- Parkinson and Parkinsonisms Center – Gaetano Pini Orthopedic Institute ASST Pini CTO (Milan) (Dr. Anna Zecchinelli: Neurologist; Dr. Nicoletta Meucci: Neurologist; Dr. Claudio Mariani: Neurologist; Dr. Giorgio Sacilotto: Neurologist; Dr. Paola Soliveri: Neurologist; Dr.ssa Michela Zini: Neurologist; Dr. Francesca del Sorbo: Neurologist; Dr. Daniela Calandrella: Neurologist Fondazione Grigioni).

Rationale

Parkinson's Disease (PD) and atypical parkinsonisms are characterized by the loss of dopaminergic neurons in the substantia nigra and other brain areas, along with the presence of Lewy neurites (LNs) and Lewy Bodies (LBs) in neurons. Atypical parkinsonisms, such as Progressive Supranuclear Palsy and Corticobasal Syndrome, are characterized by the presence of neurofibrillatory tangles composed mainly of the hyperphosphorylated form of the "Tau" protein within nerve cells. The pathology starts at least 20 years before the onset of symptoms: this is called prodromal phase. Therefore, there is an urgent need to identify early, specific, and easily accessible disease biomarkers. The presence of "Idiopathic REM sleep Behavior Disorder" (RBD), characterized by verbalizations an movements (e.g. waving, hitting, kicking) during REM sleep, can precede the onset of PD by about 15-20 years. Hence, studying this patient population is crucial for identifying valid and reliable markers in monitoring neurodegenerative progression and conversion to established PD.
The major component of LNs and LBs is the aggregated cytotoxic form of the presynaptic protein α- synuclein (α-Syn). The processes by which α-Syn begins to aggregate and causes neurotoxicity are not yet fully

understood. Apart from LBs, α-Syn accumulates in the axons and presynaptic terminals of neurons, suggesting that these accumulations act as triggers for the synaptopathy observed in PD. At the presynaptic level, α-Syn is physiologically available in its native soluble monomeric form (Burrè, 2015), where it interacts with the SNARE protein complex (consisting of VAMP-2, SNAP-25, and syntaxin-1). SNARE is assembled thousands of times per minute at the presynaptic level, enabling the exocytosis process of synaptic vesicles containing neurotransmitters. Although the physiological function of α-Syn is not fully understood, it is known to act as a chaperone in promoting SNARE assembly through a direct binding with VAMP-2 and the phospholipids present on synaptic vesicles (Bridi et al., 2018).

The term extracellular vesicles (EVs) refers to exosomes and microvesicles, i.e., nanoparticles delimited by a bilayer of phospholipids that differ in both size and origin from the cell. EVs are involved in intercellular communication by transporting bioactive molecules such as proteins and nucleic acids from the originating cell to recipient cells, where they exert their biological function. They can be isolated from almost all biological fluids, including blood, urine, saliva, seminal fluid, amniotic fluid, synovial fluid, cerebrospinal fluid, and breast milk (Urbanelli et al., 2013). Their small size in the nanometer range allows them to cross the blood-brain barrier in both directions (Wiklander et al., 2015; Bala et al., 2013; Chen et al., 2016). A recent method also used by our group (Goetzl et al., 2016), allows the isolation of neural derived extracellular vescicles (NDEVs) from peripheral blood using an antibody directed against L1CAM (CD171), a membrane protein expressed by neurons. Isolated NDEVs can then be analyzed in their content to identify easily accessible peripheral biomarkers indicative of central nervous system (CNS) pathologies.
Our group has demonstrated the presence of SNAP-25 protein in NDEVs. Quantification of SNAP-25 in NDVEs was performed in patients with Alzheimer's Disease (AD) and Healthy Control subjects (HC), showing that AD patients have reduced concentrations of SNAP-25 compared to HC (Agliardi et al., 2019). These findings are consistent with post-mortem measurements of SNAP-25 in the brain, where reduced concentrations of SNAP-25 are indeed observed in AD patients compared to healthy individuals. This suggests that the measurement of SNAP-25 in NDEVs could serve as an easily accessible index of synaptic integrity, reflecting what is happening at the cerebral level during pathology.
99% of α-Syn detected in blood originates from erythrocytes and platelets (Shi et al., 2010). Therefore, the evaluation of only neuron-derived α-Syn throughout the analysis of NDEVs is of particular interest. To date, only a few studies have investigated α-Syn in NDEVs, and results are contradictory. Shi et al. correlated monomeric α-Syn with disease severity (Shi et al., 2014). Zhao et al. reported higher levels of monomeric α-Syn in PD patients compared to controls (Zhao et al., 2018). A recent study investigated oligomeric α-Syn in NDEVs and found lower levels in PD patients compared to controls. It should be noted that the PD patients in this study were all at an early stage of the disease (Si et al., 2019).

Objectives

- **Primary Objective:** identification of diagnostic and prognostic peripheral biomarkers in PD, individuals with REM sleep Behavior Disorder, and atypical parkinsonisms. These biomarkers should be measurable in neural extracellular vescicles (EVs) of dopaminergic and serotoninergic origin, as well as glial origin.
- **Secondary Objective:** assessment of the predictive factor of these biomarkers in the response to rehabilitative treatment and the evaluation of its effectiveness in PD.

Translational productivity of clinical data

For PD, REM sleep Behavior Disorder, and other atypical parkinsonisms, there is an urgent need to identify specific, early, valid, and easily accessible disease biomarkers. Clinical symptoms are often nonspecific and late, and interventions, both clinical and pharmacological, as well as rehabilitative treatments, ideally should commence as soon as symptoms manifest, or preferably when biomarkers can confirm a seemingly silent disease to prevent or reduce irreversible damage to synapses and neurons. Oligomeric α-Synuclein (α-Syn), phosphorylated tau protein, and SNARE complex proteins in peripheral neural exosomes could potentially serve as early disease biomarker candidates. Moreover, these biomarkers could act as reliable "peripheral" indicators of the accumulation of neurotoxic α-Syn, ultimately serving as "efficacy parameters" in rehabilitative treatments for PD at different stages of severity.
The main rehabilitative outcomes correlated with biomarkers will include changes in scores on major motor scales (e.g. UPDRS), non-motor scales (e.g., non-motor symptoms scale) and neuro-psychobehavioral assessments (e.g., MoCA Test, Mini-Mental Parkinson).

Methodology

Study Design

The study will be divided into two parts: the first part of the study is an observational/cross-sectional multicenter design, involving the collection of blood samples from subjects with Parkinson's Disease (PD), atypical parkinsonisms, and REM sleep Behavior Disorder (RBD). The second part of the study, conducted entirely at the Santa Maria Nascente Center of the Don Gnocchi Foundation, is an experimental clinical trial. It will involve subjecting individuals with PD to rehabilitative treatment (see "procedures in detail").

Observational study type

Multicenter, cross-sectional study.
Participants will be recruited from the following centers:

1) II level Parkinson Outpatient Clinic; Complex Ambulatory Macro-activity (MAC); Neurorehabilitation Unit IRCCS Santa Maria Nascente della Don Gnocchi Foundation, Milan.
2) Unit of Neurology, Neurophysiology, Neurobiology; Department of Medicine; Campus Bio-Medico University of Rome (Dr. Massimo Marano; Neurologist).
3) IRCCS Ca' Granda Ospedale Maggiore Policlinico Foundation - Department of Neurosciences and Mental Health - Neurology Unit - (Dr. Alessio Di Fonzo; Neurologist).
4) University Hospital of Cagliari - Complex Operative Unit Neurology - Sleep Center (Prof. Monica Puligheddu: Neurologist; Dr. Mario Meloni: Neurologist, PhD).
5) University Hospital of Padua; Department of Neuroscience - Neurological Clinic (Prof. Miryam Carecchio; Neurologist; Prof. Angelo Antonini; Neurologist).
6) Parkinson's Center and Parkinsonisms - Gaetano Pini Orthopedic Institute-ASST Pini-CTO (Milan) (Dr. Anna Zecchinelli; Neurologist; Nicoletta Meucci, Neurologist; Claudio Mariani, Neurologist; Giorgio Sacilotto, Neurologist; Paola Soliveri, Neurologist; Michela Zini, Neurologist; Francesca del Sorbo, Neurologist; Daniela Calandrella, Neurologist - Grigioni Foundation).
7) Each participating subject who has signed the Informed Consent will undergo a blood draw at their respective Enrollment Center; subsequently, the blood samples will be processed at the Molecular Medicine and Biotechnology Laboratory of the IRCCS Santa Maria Nascente of Don Gnocchi Foundation.

Experimental study type

Monocentric, controlled, and randomized study.
Participants will be recruited at the following Center:
II level Parkinson Outpatient Clinic; Complex Ambulatory Macro-activity (MAC); NeuroRehabilitation Unit IRCCS Santa Maria Nascente of Don Gnocchi Foundation, Milan.

Experiments will be conducted on blood samples from participating subjects and processed at the Molecular Medicine and Biotechnology Laboratory of the Don Gnocchi Foundation.
Enrolled patients will undergo rehabilitative treatments (see "Procedures").

Cases/controls

In order to participate in the study, all enrolled subjects must have signed an informed consent approved by the Ethics Committee of the Don Gnocchi Foundation and by the Ethics Committees of the respective participating Centers.

We aim to enroll:

- 60 patients with PD (alpha-synucleinopathy);
- 80 patients with Progressive Supranuclear Palsy (PSP)/Corticobasal Syndrome (CBS) (Taupathy);
- 60 patients with Multisystem Atrophy (MSA) (alpha-synucleinopathy);
- 30 patients with REM sleep Behavior Disorder (alpha-synucleinopathy);
- 40 Healthy Control subjects (HC), comparable in age and sex to the recruited patients.

PSP/CBS/MSA patients' blood samples should be received from the following Centers:

- Unit of Neurology, Neurophysiology, Neurobiology; Department of Medicine; Campus Bio-Medico University of Rome (Dr. Massimo Marano: Neurologist);
- Fondazione IRCCS Ca' Granda Ospedale Maggiore Policlinico - Department of Neurosciences and Mental Health - Neurology Unit (Dr. Alessio Di Fonzo: Neurologist);
- University Hospital of Padova; Department of Neuroscience - Neurological Clinic (Prof. Miryam Carecchio: Neurologist; Prof. Angelo Antonini: Neurologist);
- UOC Parkinson Center and Parkinsonisms - Gaetano Pini Orthopedic Institute - ASST Pini - CTO (Milan) (Dr. Anna Zecchinelli: Neurologist; Dr. Nicoletta Meucci: Neurologist; Dr. Claudio Mariani: Neurologist; Dr. Giorgio Sacilotto: Neurologist; Dr. Paola Soliveri: Neurologist; Dr.ssa Michela Zini: Neurologist; Dr. Francesca del Sorbo: Neurologist; Dr. Daniela Calandrella: Neurologist Fondazione Grigioni).

A total of 20 blood samples from patients with PSP/CBS and 15 blood samples from patients with MSA should be collected by each Center.

Blood samples from patients with RBD should be collected by the following Center:

- University Hospital of Cagliari - Complex Operative Unit Neurology - Sleep Center (Prof. Monica Puligheddu: Neurologist; Dr. Mario Meloni: Neurologist, PhD).

Procedures

A peripheral blood sample (37 ml) will be collected from each study participant at T0 (enrollment), T1 (after the rehabilitation or self-tratment program) and at T2 (3 month after the end of the tratments) using a serum separator tube. Samples were allowed to clot for 30 min at room temperature and then centrifuged for 10 minutes at 1500g. After centrifugation samples were aliquoted and stored at -80°C until use. NDEVs will be enriched using a two-step method as previously shown (Mustapic M et al, 2017). Briefly, in the first step total exosomes were isolated from 500 ul of serum with ExoQuick® (System Biosciences, LCC, USA) according

to manufacturer's instructions. In the second step NDEVs were selectively enriched by immunocapture with biotinylated L1CAM (CD171) (eBio5G3, eBioscience™) antibody. Thus, proteins will be extracted from the isolated NDEVs and will be used in ELISA assays for protein dosages of oligomeric α-Syn, SNAP-25, syntaxin-1, VAMP-2, phosphorylated Tau). Western-blotting experiments will also be performed to evaluate the interactions between oligomeric α-Syn and the proteins of the SNARE complex. In addition, efforts will be made to develop a new method to be able to isolate extravesicles from dopaminergic/serotonergic neurons and glial cells by original cells' specific antibodies.

The second part of the study (interventional study) will be conducted exclusively at the IRCCS Santa Maria Nascente of the Don Gnocchi Foundation. All subjects enrolled at the Parkinson Outpatient Clinic of the Don Gnocchi Foundation who meet to the inclusion criteria, after reading the Information Sheet and signing the Informed Consent, will undergo baseline assessment and blood sampling (T0) and will then be randomly assigned to an experimental group and to a control group using a pre-filled randomization list

The experimental group will undergo an intensive, multidisciplinary rehabilitation program for the recovery and improvement of motor and "non-motor" functions with emphasis on cognitive rehabilitation.

The control group will follow a home-based self-treatment program.

Procedures in detail

Experimental rehabilitation treatment

The rehabilitation setting of the experimental group will be the traditional one in the Parkinson Outpatient Clinic system (i.e. MAC). The rehabilitation program will last for 6 consecutive weeks. The rehabilitation treatment will involve 30 sessions/5 days a week lasting 160'/day each (80' motor rehabilitation; 40' cognitive rehabilitation and 40' speech therapy) for 3 days a week and 180'/day (80' motor rehabilitation; 60' cognitive rehabilitation and 40' speech therapy) for 2 days a week. Motor rehabilitation will involve 18 sessions (3 times a week) of aerobic work on treadmill (20 minutes), balance exercises and functional reinforcement (20 minutes).

The remaining motor rehabilitation sessions will be of conventional type comprising exercises defined by the therapist according to the patient's therapeutic needs. Cognitive rehabilitation or enhancement treatment will involve an individualized program developed and supervised by a neuropsychologist; such treatment will mainly target frontal/executive/attentive/visuo-spatial-constructive functions in addition to mnestic and language functions; it will be offered both in a traditional mode (3 times/week) and through involvement in activities conducted with the support of the semi-immersive virtual reality "Virtual Reality Rehabilitation System" (VRRS) (2 times/week); VRRS uses interactive stimuli to provide the opportunity to perceive and interact, in the most real way possible, with a virtual stimulus or object, and to have immediate feedback from different sensory pathways (visual, auditory, proprioceptive, vestibular and in some cases even tactile). VRRS treatment is structured in 2 sessions per week lasting 60 minutes each for 6 consecutive weeks, for a total of

12 sessions. Specifically, VRRS treatment includes exercises designed to improve executive functions, visuospatial skills, attention, and memory (for more information on the instrumentation, see the manufacturer's website at the following link: httpp://khymeia.com/products/vrrs/).
Subjects in the experimental group will undergo an individualized program supervised by a speech therapist that will include assessment and treatment of voice (dysphonia), articulation (dysarthria), and deglutition (dysphagia). The evaluation will consist of instrumental assessment, self-assessment, and clinical assessment. Innovative techniques will be used for both assessment (acoustic speech analysis) and for treatment (biofeedback with Vitalstim). In addition, recommendations for proper deglutition will be provided, and counseling sessions (e.g., training about the use of ad hoc products for dysphagia) will be offered to the patient and caregiver. Individuals without deficits will be involved in brief training to prevent voice and deglutition disorders.

Control rehabilitation treatment
Subjects in the control group will undergo a 40'/day home-based self-treatment program consisting of stretching and active mobilization exercises. Before starting the program, subjects will undergo an educational and training session with the physical therapist to learn the correct way to perform the proposed exercises. Subjects will receive detailed exercises instructions and a diary where exercises performed, any side effects and/or specific difficulties ran into the program should be noted. Adherence to treatment will be monitored once a week by telephone contact.

Subjects in the experimental group and control group will undergo assessments and blood sampling before starting treatment (T0), at the end of treatment (T1) and 3 months after the end of treatment (T2).

Molecular investigations

- Measurement of concentrations of oligomeric α-Syn and SNARE complex proteins (SNAP-25, syntaxin-1, VAMP-2) in peripheral extracellular vesicles of neural origin (NDEs) isolated from blood samples of patients at different stages of disease and healthy controls;
- Evaluation of molecular interactions between oligomeric α-Syn and SNARE complex proteins in NDEs;
- Development of a method able to isolate from serum extracellular vesicles originating from dopaminergic neurons and glia/microglia cells by specific antibodies;
- Development of a method to be able to isolate in serum extracellular vesicles originating from dopaminergic neurons and from serotonergic and noradrenergic neurons by specific antibodies;
- Measurement of oligomeric α-Syn and SNARE complex protein concentrations in NDEs before and after rehabilitation treatments;

- Study of the correlation between the levels of oligomeric α-Syn in NDEs and the two main clinical forms of PD: akinetic-rigid and tremor-dominant.
- Measurement of the concentration of oligomeric α-Syn and phosphorylated Tau in neural/glial exosomes and study of their correlation in PD, atypical parkinsonisms (multisystem atrophy; progressive supranuclear palsy and cortico-basal syndrome) and RBD and evaluation of the potential role in differential diagnosis;
- Study of the correlation between oligomeric α-Syn of neural origin (dopaminergic neurons), oligomeric α-Syn of microglial origin, and possible changes at various stages of disease.

Clinical, motor, and functional assessments

Measurement of main clinical parameters (motor and non-motor symptomatology) at timepoints T0, T1 and T2 using scales and questionnaires specific to PD and atypical parkinsonisms:

1) Neurological and neuro-psycho-pathological clinical assessments:

A) Neurological assessments:

- Anamnestic information (type and date of onset; date of diagnosis; presence/description of instrumental exams; current signs and symptoms);
- Current drug therapy and any changes (drug therapy change dates, dosages -for ledd calculation- and drug therapy intake times);
- Information collection for compilation of the following scales:
  - Modified Hoehn and Yahr Scale (Modified H&Y) (Goetz et al. 2004);
  - Movement Disorders Society Unified Parkinson's disease rating scale Part I-IV (MDS-UPDRS Part I-IV) (Antonini et al. 2013);
  - Non-Motor Symptoms Scale (NMSS) (Chaudhuri et al., 2007; Cova et al., 2017);
  - Composite Autonomic Symptoms Score COMPASS 31 (Pierangeli et al. 2015);
  - Modified Barthel Index (MBI) (Galeoto et al.2015);
  - Modified Cumulative Illness Rating Scale (CIRS) (Salvi et al., 2008);
  - Numeric Rating Scale (NRS) (Hartrick et al., 2003);
  - 39-item Parkinson's Disease Questionnaire (PDQ-39) (Galeoto et al., 2018);
  - Parkinson Fatigue Scale (PFS) (Brown et al., 2005; Siciliano et al., 2019);
  - Barratt Impulsivenes Scale-11 (BIS-11) (Fossati et al., 2001);
  - Questionnaire for Impulsive-Compulsive Disorders in Parkinson's disease (QUIP-RS-IT) (Weintraub et al., 2009);
  - Pittsburgh Sleep Quality Index (PSQI) (Curcio et al., 2013);
  - Parkinson Sleep Scale (PSS) (Pellecchia et al 2012);
  - Epworth Sleepiness Scale (ESS) (Vignatelli et al. 2003);

- REM sleep behavior disorder screening questionnaire (RBDSQ) (Marelli et al., 2016).

B) Neuropsychological and psycho-behavioral assessments:

1) I Level - cognitive tests:

- Montreal Cognitive Assessment (MoCA Test) (Conti et al., 2014; Santangelo et al., 2014);
- Test del Breve Racconto (vers. Babcock '6 Dicembre'; Carlesimo et al., 2002);
- Trail Making Test (TMT) (Giovagnoli et al., 1996);
- Raven Coloured Progressive Matrices (CPM-47) (Caltagirone et al, Carlesimo et al, 1996);
- Forward and Backward Verbal Span (Monaco et al., 2013);
- Copy and Recall of Rey-Osterrieth Complex Figure (Caffarra et al., 2002);
- Alternate Verbal Fluency (Costa et al., 2014);
- Frontal Assessment Battery (FAB) (Appollonio et al., 2005);
- Stroop Test-Short Version (Caffarra et al., 2002);
- Mini-Mental Parkinson (MMP) (Costa et al., 2013);
- Boston Naming Test (shortened version) (Mack et al., 1992).

2) II Level - Neuropsychiatric/functional/Quality of Life (QoL) scales (some completed by the patient, others administered and completed by the caregiver):

- Cognitive Reserve Index-questionnaire (CRI-q) (Nucci et al., 2011);
- Beck Depression Inventory-II (BDI-II) (Beck et al., 1996; Ghisi et al., 2006) (filled by patient);
- State-Trait Anxiety Inventory. Form Y (STAI-Y) (Spielberger et al., 1983; Pedrabissi & Santinello, 1989) (completed by patient);
- Index of Autonomy in Activities of Daily Living (ADL) (Katz et al., 1963) (completed by caregiver);
- Index of autonomy in Instrumental Activities of Daily Living (IADL) (Lawton et al., 1969) (completed by caregiver);
- Functional Activities Questionnaire (FAQ) (Pfeffer et al., 1982; vers. Sansotera e Foderaro, 2007) (completed by caregiver);
- Caregiver Burden Inventory (CBI) (Novak e Guest, 1989) (completed by caregiver);
- NeuroPsychiatric Inventory Questionnaire (NPI-Q) (Kaufer et al., 2000) (completed by caregiver).

3) II Level - cognitive tests

- Clock Drawing Test (CDT) (Mondini et al., 2003);
- Free and Cued Selective Reminding Test (FCSRT) (Frasson et al., 2011);
- Forward e Backward visuo-spatial span (Corsi Test) (Monaco et al., 2013);

- Attentive Matrices (Spinnler e Tognoni, 1987);
- Symbol Digit Modalities Test (SDMT) (Vers. Orale) (Nocentini et al., 2006);
- Gesture Imitation Test (vers.: De Renzi et al., 1968; standardization: De Renzi et al., 1980, 1986);
- Weigl's Sorting Test (version e standardization: Spinnler e Tognoni, 1987).

4) II Level - Neuropsychiatric/functional/Quality of Life (QoL) scales (some completed by the patient, others administered and completed by the caregiver):

- Dimensional Apathy Scale (I-DAS) (Santangelo et al., 2017) (completed by patient);
- Snaith-Hamilton Pleasure Scale (SHAPS) (Santangelo et al., 2009) (completed by patient);
- Toronto Alexithymia Scale (TAS-20) (Taylor et al., 1992; Bressi et al., 1996).

C) Motor and functional assessments [motor assessments will be administered about an hour after the first daily intake of the dopaminergic drug (levodopa; dopamine agonist etc)]:

- Timed-Up and -Go Test (TUG) to assess dynamic balance
- Modified Dynamic Gait Index (mDGI) to assess dynamic balance;
- 6 Minute Walk test (6MWT) to detect resistance in walking;
- 10-meter walk test to assess walking speed;
- 5-time sit-to-stand to assess lower limb strength;
- Retrospective collection of the number of falls in the past 2 months;
- Gait analysis by optoelectronic system; subjects will walk continuously within the laboratory until they reach a perceived fatigue state of 17 ("very heavy" exertion) on BORG's modified scale. Fatigue state will be monitored every minute to investigate changes in walking-related parameters related to fatigue.

Instrumental assessment by wearable device (wearable):

The experimental and the control groups will undergo actigraphic monitoring in the week before treatment begins (T0), in the week after treatment ends (T1), and during the three-month follow-up (T2). Subjects will wear an actigraph at each wrist with the aim of collecting quantitative information on motor activity. Each subject during these days will be required to write a diary of motor habits and activities and the pharmacological therapy The actigraph, which looks like a bracelet/wristwatch (https://www.geneactiv.org/actigraphy/geneactiv- original/presente marchiatura CE 2004/108/EC; tested to BS EN 61000-6-1 :2007 and BS EN 61000-6-3 :2007) illustrated below, allows the automatic recording of some informations, which can then be downloaded, extracted and processed according to algorithms defined by an engineering level through a connection to a PC, in order to obtain, for example, graphs representing the alternation of activity/rest, possible sleep disorders, tremor (present/absent, unilateral/bilateral), and other

information relating to motor activity. In order to calibrate the actigraphic method (Rabuffetti et al. 2016) to individuals with PD, it will be developed a laboratory trial, that includes predefined and controlled motor tests (such as straight walking at different speeds, upper limb ergometer operation in the absence of an applied load at a predefined and controlled speed, standardized functional tests such as the TUG test). It is expected that this pilot trial will involve no less than three persons with compatible characteristics with recruitment in the main study.

To supervise adherence and active participation in the rehabilitation treatment, both the subjects in the control group and the subjects in the experimental group will be assessed the Italian version of the 'Pittsburgh Rehabilitation Participation Scale (PRPS)' (Iosa et al. 2021). The administration will be carried out once a week both to the subjects in the control group and to the subjects in the experimental group.

Recruitment of participants

All subjects must fall within the age range of 50 to 85 years. Enrolled subjects will undergo assessments using scales for staging disease severity and evaluations of motor and non-motor functions (sleep disturbances, dysautonomia, cognitive-behavioral disorders). A comprehensive pharmacological history will be obtained from all enrolled subjects. Accordingly, levodopa equivalent dose will be calculated for each subject according to Tomlinson criteria (Tomlinson et al., 2010).
Control subjects will have to be comparable in age and gender to patients, with no cognitive impairments or evidence of acute or chronic neurological conditions at the time of enrollment. Control subjects will be selected among caregivers.
PD diagnosis will be performed by neurologists following the "MDS clinical diagnostic criteria for Parkinson's disease" (Postuma et al., 2015). Subjects with PD will be included when they will fit a staging of disease severity (according to Hoehn & Yahr) between 1 and 3.
The diagnosis of Progressive Supranuclear Palsy (PSP) will be made according to the criteria of the Movement Disorders Society (Movement Disorder Society-endorsed PSP Study Group, 2017).

The diagnosis of Corticobasal Syndrome (CBS) will follow the Armstrong criteria of 2013 (Armstrong MJ et al., 2013).
The diagnosis of Multiple Systemic Atrophy (MSA) will be based on the Gilman criteria from 2008 (Gilman et al., 2008).
The diagnosis of REM Sleep Behavior Disorder (RBD) will be made according to the criteria of the "International Classification of Sleep Disorders, Third Edition (ICSD-3)".
The initial sample size of 80 subjects with PSP and CBS, 60 with MSA, and 30 with RBD is based on an approximate estimate of the number of patients typically seen at the participating centers.

1) Neurology, Neurophysiology, Neurobiology Unit; Department of Medicine; Campus Bio-Medico University of Rome (Dr. Massimo Marano, Neurologist).
2) IRCCS Ca' Granda Ospedale Maggiore Policlinico Foundation - Department of Neuroscience and Mental Health - Neurology Unit - (Dr. Alessio Di Fonzo, Neurologist).
3) University Hospital of Cagliari - Complex Neurology Unit - Sleep Center (Prof. Monica Puligheddu, Neurologist; Dr. Mario Meloni, Neurologist, PhD).
4) University Hospital of Padua; Department of Neuroscience - Neurological Clinic (Prof. Miryam Carecchio, Neurologist; Prof. Angelo Antonini, Neurologist).
5) Parkinson and Parkinsonism Center - Gaetano Pini Orthopedic Institute-ASST Pini-CTO (Milan) - (Dr. Anna Zecchinelli, Neurologist).

At the actual state of the art, there are no clinical efficacy studies on rehabilitative treatment for the biomarkers under indvestigation (e.g., α-synuclein, SNARE protein complex). Thus, the sample size was calculated based on preliminary results from a cross-sectional study (Agliardi et al., 2021). The just-mentioned study was conducted on 32 patients recruited within the PD center of Don Gnocchi Foundation. Thus, considering 2 degrees of freedom, a power of 80%, $\alpha=5\%$ and a dropout rate of 20%, 36 patients per study group are required, for a total of 72 patients.

Statistical analyses

Enrolled subjects must be comparable in terms of age and gender. The normality of continuous variable distributions will be assessed using the Shapiro-Wilk and Kolmogrov-Smirnov tests. Group comparisons will be conducted using the t-student and ANOVA test for normally distributed data, and Kruskal-Wallis and Mann-Whitney tests for non-normally distributed data. The possible validity of the measurement of the aforementioned exosomal proteins as biomarkers for PD will be evaluated through Receiver Operating Characteristic (ROC) analysis. This involves the calculation of the area under the curve (AUC), sensitivity, specificity, and a 95% confidence interval. A significance level of $p \leq 0.05$ will be considered in all cases. Logistic regression analyses will be employed to explore associations between independent variables (demographic variables, parkinsonisms phenotypes including motor and nonmotor scales, staging of disease severity, LEDD, and rehabilitative outcomes) and the dependent variable (serum biomarkers - α-Syn oligomeric and SNARE complex proteins)

Challenges

The most significant challenge may arise from the low adherence to the protocol due to patients' non-consent. In this regard, a check of numerosity will be conducted at the end of the first year. There are no criticisms

associated with the use of the instruments (actigraphs) as commercially available CE-marked devices and measurement systems for everyday use will be employed.

Expected results

We expect to find that the concentrations of α-Syn oligomers, phosphorylated tau protein, and SNARE complex proteins (and their interrelationships), measured in extracellular vesicles of cerebral origin isolated from peripheral blood, may serve as biomarkers for MSA, RBD and atypical parkinsonisms. In addition, the potential correlation with clinical and instrumental data, along with rehabilitative outcomes, will allow for the assessment of the predictive role of the examined biomarkers and their possible utilization as 'efficacy parameters' in rehabilitation for PD. Specifically, it is expected that rehabilitative treatment may modulate the concentrations of α-Syn oligomers and SNARE complex proteins.

Project Phases

	**Timing**		
**Activity**	**From**	**To**	**Responsible**
Patient Enrollment and Biological Sample Collection	January 2020	June 2024	Francesca Lea Saibene; Pietro Arcuri; Anna Castagna; Elisabetta Farina; Margherita Alberoni; Elena Calabrese; Anna Salvatore
Data Tabulation and Intermediate Statistical Analyses	February 2020	September 2023	Franca Guerini; Cristina Agliardi
Molecular Biology analyses	February 2020	September 2024	Cristina Agliardi
Abstract writing to participate in congresses	June 2020	December 2024	Francesca Lea Saibene; Mario Meloni; Franca Guerini; Cristina Agliardi
Data Tabulation and Final Statistical Analyses	March 2021	September 2024	Francesca Lea Saibene; Franca Guerini; Cristina Agliardi; Marco Rabuffetti (per attività actigrafica)
Paper writing	September 2020	December 2024	Francesca Lea Saibene; Mario Meloni; Pietro Arcuri; Franca Guerini; Cristina Agliardi; Mario Clerici

Economic aspects

The study was previously approved by the Scientific Direction of the Foundation as part of the Current Research activities scheduled for the year 2020. Consequently, all costs associated with the execution of the study (e.g., materials for sample collection, costs of blood sample shipments) will be covered by the funds allocated by the Ministry of Health to the Don Gnocchi Foundation for the aforementioned Current Research activity.

REFERENCES

A.A. of S. Medicine, International Classification of Sleep Disorders 3nd edition. Diagnostic and coding manual, 2014.

Agliardi C, Guerini FR, Zanzottera M, Bianchi A, Nemni R, Clerici M. SNAP-25 in Serum Is Carried by Exosomes of Neuronal Origin and Is a Potential Biomarcatore of Alzheimer's Disease. Mol Neurobiol. 2019Aug;56(8):5792-5798. doi:10.1007/s12035-019-1501-x.

Agliardi C, Meloni M, Guerini FR, Zanzottera M, Bolognesi E, Baglio F, Clerici M. Oligomeric alpha-Synand SNARE complex proteins in peripheral extracellular vesicles of neural origin are biomarkers

for Parkinson's disease. Neurobiol Dis. 2021 Jan;148:105185. doi: 10.1016/j.nbd.2020.105185. Epub 2020Nov 18.

Armstrong MJ, Litvan I, Lang AE, et al. Criteria for the diagnosis of corticobasal degeneration. Neurology. 2013;80(5):496-503.

Bala S, Csak T, Momen-Heravi F, Lippai D, Kodys K, Catalano D, Satishchandran A, Ambros V et al (2015) Biodistribution and function of extracellular miRNA-155 in mice. Sci Rep5:10721. https://doi.org/10.1038/srep10721

Bridi JC, Hirth F. Mechanisms of α-Synuclein Induced Synaptopathy in Parkinson's Disease. Front Neurosci. 2018 Feb 19;12:80. doi:10.3389/fnins.2018.00080.

Burré J. The Synaptic Function of α-Synuclein. J Parkinsons Dis. 2015;5(4):699-713. doi: 10.3233/JPD-150642.

Chen CC, Liu L, Ma F, Wong CW, Guo XE, Chacko JV, Farhoodi HP, Zhang SX et al (2016) Elucidation of exosome migration across the blood-brain barrier model in vitro. Cell Mol Bioeng 9(4):509– 529. https://doi.org/10.1007/s12195-016-0458-3.

Gilman S, Wenning GK, Low PA, Brooks DJ, Mathias CJ, Trojanowski JQ, Wood NW, Colosimo C, Durr A, Fowler CJ, Kaufmann H, Klockgether T, Lees A, Poewe W, Quinn N, Revesz T, Robertson D, Sandroni

P, Seppi K, Vidailhet M. Second consensus statement on the diagnosis of multiple system atrophy.Neurology. 2008;71:670–676.

Goetzl EJ, Kapogiannis D, Schwartz JB, Lobach IV, Goetzl L, Abner EL, Jicha GA, Karydas AM, Boxer A,Miller BL. Decreased synaptic proteins in neuronal exosomes of frontotemporal dementia and Alzheimer's disease. FASEB J. 2016 Dec;30(12):4141-4148.

Höglinger GU, Respondek G, Stamelou M, Kurz C, et al. Movement Disorder Society-endorsed PSPStudy Group. Clinical diagnosis of progressive supranuclear palsy: The movement disorder society criteria. Mov Disord. 2017 Jun;32(6):853-864.

Iosa M, Galeoto G, De Bartolo D, Russo V, Ruotolo I, Spitoni GF, Ciancarelli I, Tramontano M, AntonucciG, Paolucci S, Morone G. Brain Sci. 2021 May 13;11(5):626. doi: 10.3390/brainsci11050626

Mustapic M, Eitan E, Werner JK Jr, Berkowitz ST, Lazaropoulos MP, Tran J, Goetzl EJ, Kapogiannis D. Plasma Extracellular Vesicles Enriched for Neuronal Origin: A Potential Window into Brain Pathologic Processes. Front Neurosci. 2017 May 22;11:278. doi: 10.3389/fnins.2017.00278.

Postuma RB, Berg D, Stern M, MDS clinical diagnostic criteria for Parkinson's disease. Mov Disord. 2015 Oct;30(12):1591-601. doi: 10.1002/mds.26424.

Rabuffetti M, Meriggi P, Pagliari C, Bartolomeo P, Ferrarin M. Differential actigraphy for monitoring asymmetry in upper limb motor activities. Physiol Meas. 2016 Oct;37(10):1798-1812. doi: 10.1088/0967-3334/37/10/1798.

Shi M, Zabetian CP, Hancock AM, Ginghina C, Hong Z, Yearout D, Chung KA, Quinn JF, Peskind ER, Galasko D, Jankovic J, Leverenz JB, Zhang J. Significance and confounders of peripheral DJ-1 and alpha-synuclein in Parkinson's disease. Neurosci Lett. 2010 Aug 9;480(1):78-82. doi: 10.1016/j.neulet.2010.06.009.

Si X, Tian J, Chen Y, Yan Y, Pu J, Zhang B. Central Nervous System-Derived Exosomal Alpha-Synuclein in Serum May Be a Biomarcatore in Parkinson's Disease. Neuroscience. 2019 Aug 10;413:308-316. doi: 10.1016/j.neuroscience.2019.05.015.

Tomlinson CL, Stowe R, Patel S, Rick C, Gray R, Clarke CE. Systematic review of levodopa dose equivalency reporting in Parkinson's disease. Mov Disord. 2010 Nov 15;25(15):2649-53.

Urbanelli L, Magini A, Buratta S, Brozzi A, Sagini K, Polchi A, Tancini B, Emiliani C (2013) Signaling pathways in exosomes biogenesis, secretion and fate. Genes (Basel) 4(2):152– 170. https://doi.org/10.3390/genes4020152

Wiklander OP, Nordin JZ, O'Loughlin A, Gustafsson Y, Corso G, Mäger I et al (2015) Extracellular vesiclein vivo biodistribution is determined by cell source, route of administration and targeting. J Extracell Vesicles4:26316. https://doi.org/10.3402/jev.v4.26316.

Zhao ZH, Chen ZT, Zhou RL, Zhang X, Ye QY, Wang YZ. Increased DJ-1 and α-Synuclein in Plasma Neural-Derived Exosomes as Potential Markers for Parkinson's Disease. Front Aging Neurosci. 2019 Jan 14;10:438. doi:10.3389/fnagi.2018.00438.

Possible benefits for the patient

The expected benefits are directed towards acquiring greater scientific knowledge about the disease. Participation in the study does not impact the patient's usual care since it does not involve the exclusion or reduction of the routine treatment.
The better description of motor, non-motor and cognitive aspects in PD and Parkinsonisms could provide useful information in diagnostic and prognostic terms for the optimization of resources in terms of (lo cambierei con: with regard to) rehabilitation and continuity of care (also through new tools and procedures - e.g., motor activity monitoring with actigraphy, also at home), with possible spillover to the NHS for nonpharmacological intervention protocols on the population in question.

Possible risks/side effects for the patient

The patient will not be exposed to any clinically relevant risks.
The only potentially critical procedure for the patient may be the blood sampling, which could cause hematoma at the site of sampling in some cases: for this reason, special attention will be paid during this phase to subjects undergoing antiplatelet and anticoagulant therapy.
No possible risks or side effects are identifiable because data recorded through actigraphs (telemonitoring of motor information) has no risks or possible side effects.

Data collection and processing

Clinical and anamnestic information will be collected for each patient such as disease duration, disease progress, past treatments, and current therapies. Scales for the assessment of motor and non-motor symptomatology will also be administered for individuals with PD.
General data (degree of kinship, gender, and age) regarding the Caregivers of the enrolled patients will be collected. The collected information related to the recruited subjects and caregivers will play a key role in the statistical analyses; therefore, it will be used as covariates during these analyses. All clinical anamnestic data, (assessments before and after rehabilitative treatments) and biological data will be collected in an anonymized

database by progressive numeric code and they will be evaluated as aggregate data accessible only by participating researchers.
